# Determinants of Intraparenchymal Infusion Distributions: Modeling and Analyses of Human Glioblastoma Trials

**DOI:** 10.3390/pharmaceutics12090895

**Published:** 2020-09-21

**Authors:** Martin Brady, Raghu Raghavan, John Sampson

**Affiliations:** 1 Therataxis, Baltimore, MD 21210, USA; mbrady@therataxis.com; 2 Department of Neurosurgery, Duke University, Durham, NC 27710, USA; John.Sampson@duke.edu

**Keywords:** CED (convection-enhanced delivery), targeted drug delivery, drug delivery planning, predictive flow models

## Abstract

Intra-parenchymal injection and delivery of therapeutic agents have been used in clinical trials for brain cancer and other neurodegenerative diseases. The complexity of transport pathways in tissue makes it difficult to envision therapeutic agent distribution from clinical MR images. Computer-assisted planning has been proposed to mitigate risk for inadequate delivery through quantitative understanding of infusion characteristics. We present results from human studies and simulations of intratumoral infusions of immunotoxins in glioblastoma patients. Gd-DTPA and ^124^I-labeled human serum albumin (^124^I-HSA) were co-infused with the therapeutic, and their distributions measured in MRI and PET. Simulations were created by modeling tissue fluid mechanics and physiology and suggested that reduced distribution of tracer molecules within tumor is primarily related to elevated loss rates computed from DCE. PET-tracer on the other hand shows that the larger albumin molecule had longer but heterogeneous residence times within the tumor. We found over two orders of magnitude variation in distribution volumes for the same infusion volumes, with relative error ~20%, allowing understanding of even anomalous infusions. Modeling and measurement revealed that key determinants of flow include infusion-induced expansion and loss through compromised BBB. Opportunities are described to improve computer-assisted CED through iterative feedback between simulations and imaging.

## 1. Introduction

*Le Président:* Vous tâcherez d’être bref. *La Brige:* Je tâcherai d’être clair. (The Judge: Make an effort to be brief. La Brige (the defendant): I shall make an effort to be clear. (From a one-act play L’article 360, available on the web, by the French author Georges Courteline, performed in 1900))

There is a vast effort to cure devastating neurological disorders. Systemic, intrathecal, and intraventricular therapies in general do not go past the blood–brain barrier, and thus a drug’s impact may be significantly reduced since only a small percentage of brain volume is treated while most of the agent is cleared with no therapeutic effect. The most likely near-term solution is intraparenchymal treatment, also termed convection-enhanced delivery (CED), where the drug is infused directly into brain tissue with a minimally invasive catheter(s). 

Experienced neurosurgeons can reliably create drug distributions in a small volume near the tip of the catheter. Our work in this area is based on the premise that one cannot infer the final distribution of a large infusion by simply looking at an MRI image prior to or early in an infusion. Wide differences in advection speeds between gray matter and white matter; the alteration of the material properties of the brain in response to the infusion itself; the shunting effect of CSF spaces including small sulcal spaces; and other shunting pathways make an intuitive prediction of the final distribution impossible. There are an interstitium response to the infusion and to the device inserted and an agent response due to the properties of the interstitium and its boundaries to the CSF and vascular spaces that have major effects on the resulting distribution. These factors result in an *unpredictability* which needs to be addressed in planning an infusion. Pharmaceutical and academic communities pursuing cures for rare diseases require evidence that widespread, robust infusions are achievable, and that individual dosage is verifiable. For these purposes, we have been developing a computational simulation procedure for planning infusions, upon which we have reported in detail in the past [1]. The purpose of this paper is threefold: (1) to describe the improvements made to the original algorithms, available commercially in BrainLab’s iPlanFlow™; (2) to describe its performance with data taken in human clinical trials at Duke University; and (3) to describe the lessons we may learn from the observations and the models about the interstitial pathways for transport of therapeutics. We begin with summarizing the simulation procedure’s salient features, and we conclude with the lessons learned. An opinion paper that summarizes our findings and speculations in this area is available [2]. Our work is both mathematical and algorithmic. We have published most of our developments, and so we refer to these publications for details, confining ourselves here for the most part to the concepts involved. 

## 2. Materials and Methods

This section is divided into two major subsections. The first deals with our approach to modeling the delivery and transport of molecules in the brain, and the second with the experimental and imaging protocol used on the human subjects. Details of the various MR imaging protocols to which we refer can be found in Brown et al. [3].

### 2.1. Simulation Models and Methods

Our earlier simulation model is described in detail in [1] and was implemented as part of the *iPlanFlow™* software of BrainLab AG, Munich, Germany. Here, we describe and evaluate a number of improvements to the original simulation model.

#### 2.1.1. Mathematical Modeling of CED

The starting point of our simulations is the basic continuum equations of fluid and solute flow in porous media as described in the seminal publication [4] on the subject of CED, and in our previous exposition of our simulation [1]. We here summarize the phenomena that need to be modeled, particularly in the light of claims on glymphatic spaces [5,6,7], although these claims have been since disputed [8,9,10]. In this paper, we do not write any equations described in our previous publications, although we do introduce equations giving details of methods that we have not yet published, such as the description of poroelasticity employed in our simulations in Section 2.1.2. We provide the references where any omitted equations are developed and described.

The simulation produces numerical solutions to two sets of equations, together with the boundary conditions involved. We wish to compute the interstitial concentration *c* of a free molecule which is injected in solution into the brain, as measured at the place *p* at the time *t*. It should therefore be understood that *c* is a function of place and time. This computation describes molecules available for therapeutic action at a defined place. Molecules that have been bound and internalized are no longer available for further transport. Molecules that are bound to cells are actively engaged in the therapy and we return to these shortly. The processes that transport the free molecules are: (i) advection; and (ii) diffusion. Processes that affect their concentration “in place” are: (iii) sinks that remove them entirely from brain parenchyma, including losses through the capillaries into the circulatory system, losses into the CSF spaces including the ventricles, and losses into the sub-arachnoid CSF due to the ubiquitous presence of sulci; (iv) irreversible degradation; and (v) binding and internalization, this last being the desired outcome. The equation describing the flow of the particle (therapeutic molecule or surrogate tracer, for example) is written

(1) $$\frac{\partial \left(\varphi c\right)}{\partial t}=-\mathrm{div}\left(vc\right)+\mathrm{div}\left(\varphi D\;\mathrm{grad}\left(c\right)\right)-kc-k_{1}\left(B-b\right)c+k_{2}b.$$

Although we write the equations describing the motion and binding of the molecules or particles in tissue, we do not pause to discuss them in any detail. We refer to [4] (see equations in Appendix A in that paper), as well as our detailed presentation on modeling [1] (Equations (1)–(4)) for a discussion of the meaning of the mathematics; see also discussion on the issues involved in modeling in [2]. For convenience, we give the meaning of the symbols in Table 1. We may write the above in words: the rate of change of *c*(*p*,*t*) is the sum of contributions from advection with fluid velocity v + diffusion + loss through capillaries (also irreversible degradation) + transformation to bound molecules *b*(*p*,*t*).

The CED infusion source also affects concentration, but this is treated as a boundary condition, as are the CSF boundaries. The mathematical form of the equations that describe these processes in an approximation that is linear in concentration are available in [4] (see equations in Appendix A in that paper), as well as in our detailed presentation on modeling [1]; see also discussion on the issues involved in modeling in [2].

The advection term involves the carrying of the molecule by its solvent fluid, and one usually uses the velocity of the fluid itself for the velocity of the transport of the molecule. (Sometimes a “retardation factor” less than one is used to model the advection of larger molecules which may encounter resistance in their transport due to being comparable in size to the interstitial widths, or due to encountering macromolecules in the interstitial spaces. We have not used such a factor in our simulations.) We compute the fluid velocity simply as Darcy flow in a porous medium, which means the flow velocity is proportional to the pressure gradient in the medium:

(2) v=−K grad(p)

which states that the velocity of fluid is proportional to the pressure gradient. We may combine this with the equation for fluid loss which is

(3) $$\mathrm{div}\left(v\right)=-\beta^{2}p$$

or, in words, the rate of loss of fluid per unit volume is minus the capillary conductance times the excess interstitial pressure difference, so that, by substituting Equation (2) into Equation (3), we obtain an equation for the pressure. Again, the cited references discuss these equations in detail.

The minus sign in Equation (2) is needed because fluid flow is oriented towards decreasing pressure. Equation (3) states that fluid volume is not conserved in brain since the infusion induces extra pressure that allows excess fluid to leak back into the capillaries. These losses are an entirely different phenomenon than the loss of drug molecule through the capillaries, owing not only to the relative sizes of water and drug molecules but also due to aquaporin channels in the capillary membranes. As in (1), the infusion and the CSF boundaries act as sources and sinks for the fluid, but these are treated as boundaries and so do not appear in the field equations. Again, the cited references contain the mathematical forms of the two previous equations, both of which are necessary to solve for the fluid velocity, and we do not make further reference to the phenomena described by (3) except briefly while discussing “glymphatics” in Section 4.1.

Returning to (2), it is crucial that the response of the tissue to the pressure gradient is *nonlinear* so that the conductivity changes as the flow penetrates into the medium, and with the flow rates (or pressure) involved. This effect is extremely significant, and noted early in [11]. The direction of the flow in response to a given pressure gradient will depend on the direction of the pressure gradient and on the properties of the medium, which in turn change in the presence of introduced fluid and increased hydrostatic pressure. These effects due to the alteration of the medium are the most important.

Finally, to close the loop, we need to account for the bound molecules which remain “in place”:

(4) $$\frac{\partial b}{\partial t}=k_{1}\left(B-b\right)c-\left(k_{2}+k_{irr}\right)b$$

meaning that the rate of change of *b*(*p*,*t*) = reverse reaction to free drug *c*(*p*,*t*) + internalization and degradation, with the bound particles staying in place.

For a detailed discussion of how binding kinetics affects the free drug concentration together with numerical results, see [12]. In this paper, we compute only the concentrations of tracers, both small and large molecules, so that no binding is introduced. All losses are posited to be linear terms in the concentration such as would obtain due to capillary losses and different sinks. The relation between tracer and active drug distributions is deferred to Section 4.

**Figure 1:**
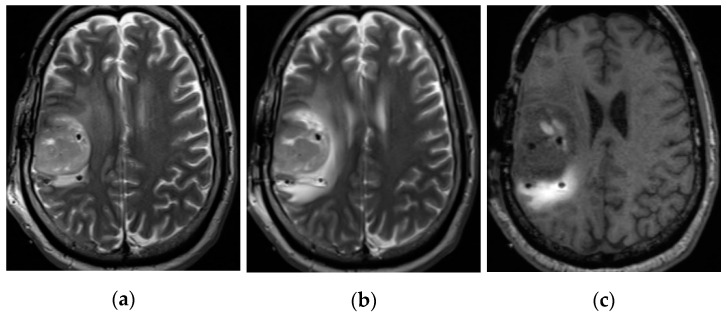
Infusion induces expansion (Axial T2-weighted images, MR1-1 Patient 8). (**a**) After 2 h of infusion, a small amount of edema (bright regions) is visible near an infusion catheter (black dots), posterior to the tumor in this T2-weighted image. (**b**) After 24 h of infusion, the size and intensity of the T2-weighted signal in the nearby white matter has increased. (**c**) T1-weighted imaging after 24 h of infusion shows the distribution of gadolinium tracer).

To understand interstitial flow in the brain, poroelasticity is key. The effects of the dynamical response of the interstitium to the introduction of fluid and molecules is far more determinative of the eventual distributions than is the geometry of the catheter or the infusion protocol (within limits). It is well known that infusions can expand the interstitium [11], making the white matter appear edematous, as illustrated in Figure 1. This increase in pore fraction correlates with the distribution of infusate, as measured using a gadolinium tracer compound. The expansion of the white matter greatly increases its hydraulic conductivity (and to a lesser extent, its diffusivity) in this region, and tends to make the tissue far less anisotropic.

**Figure 2:**
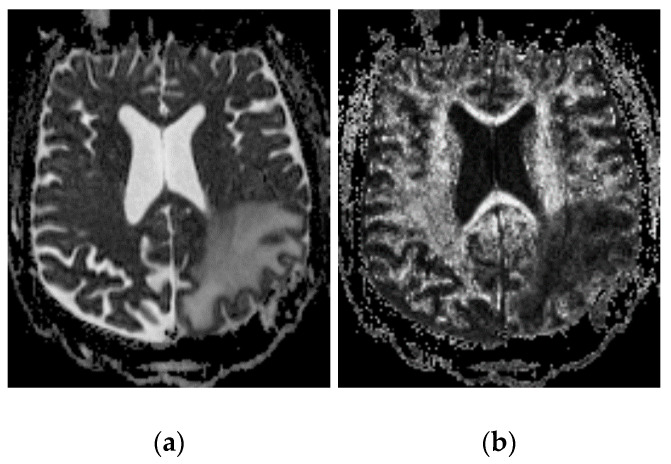
The expansion of white matter renders it isotropic (MR1-1 Patient 7): (**a**) average diffusion coefficient (ADC) map; and (**b**) fractional anisotropy (FA) map.

An example of the reduction of diffusion anisotropy is shown in Figure 2, which displays the ADC in a tumor patient with a large region of white matter edema. The diffusion coefficient has increased, but, correspondingly, the fractional anisotropy (shown as an intensity map in Figure 2 has now become quite small (compare with the contralateral region in the other hemisphere). This suggests that the well-known “channeling” of infusions in white matter tracts is primarily due to the ready expandability of the fibers, which allows the conductivity to increase very dramatically, rather than their anisotropy.

To accommodate this effect in a predictive simulation, we estimate the change in pore fraction and hydraulic conductivity for the infusion protocol and planned placement of the catheters using the linear theory of poroelasticity, as described in [13]. The previous implementation that is part of iPlanFlow™ is an *ad-hoc* procedure described in [1]. The linear theory of poroelasticity does not use the interstitial volume fraction *φ* as one of the standard variables in its formulation. However, the formulas from the standard theory may be rearranged to write the change *δφ* of this volume fraction in terms of the change *δp* in interstitial fluid pressure and the overall change in volume fraction of tissue, called dilatation and denoted *δe* as

(5) $$\delta \varphi =\frac{\left(\alpha -\varphi \right)\left(1-\alpha \right)}{B}\delta p+\left(\alpha -\varphi \right)\delta e.$$

The coefficient *α* is called the Biot–Willis coefficient (note this letter a has an entirely different meaning in this subsection from that in the previous one), and is bounded as follows,

(6) φ≤α≤1.

The coefficient *B* is called the drained bulk modulus (the symbol for the bulk modulus is usually *K*, but, in this paper, we reserve that notation for the hydraulic conductivity) and its bounds are as follows:

(7) $$0\leq B\leq \left(1-\varphi \right)K_{s},$$

where *K_s_* can be considered to be the usual bulk compressibility modulus of the solid component of the tissue. Equation (7) is not the most transparent way to develop the linear theory of poroelasticity, but it is the best starting point for estimating the increase of interstitial fraction upon infusion. A suspension of solid particles in fluid would have *B =* 0, so that such a “material” will in fact expand without any applied stress, expanding just with the introduction of fluid into the volume In fact, *B*, which is the compressibility of tissue with the fluid in the interstitial spaces allowed to flow freely, is usually quite small. In white matter, with even substantial increase in fluid content, *B* is a good approximation to a suspension.

In the full theory of poroelasticity, the canonical variables *δp* and *δe* are coupled and have to be solved together, requiring more equations. However, in certain circumstances, these variables may be decoupled. If we assume an isotropic elasticity of the tissue framework and assume that the pressure at the boundaries of the brain does not change during the infusion, it may be shown that the dilatation and the pressure are proportional:

(8) $$\delta e=\frac{\alpha }{K_{V}}\delta p$$

The coefficient $K_{V}$ in turn is defined by the equation

(9) $$K_{V}^{-1}=B^{-1}\frac{1+\nu }{3\left(1-\nu \right)}$$

where ν is called the drained Poisson ratio. In the limit where the drained Poisson ratio is ½ (incompressibility under drained conditions), $K_{V}=K$. However, more generally, we find

(10) $$\delta \varphi =\left(\alpha -\varphi \right)\left[1-\alpha \left(1-\frac{1+\nu }{3\left(1-\nu \right)}\right)\right]\frac{\delta p}{B}$$

The expression for ν is

(11) $$\nu =½\frac{3B-2G}{3B+G}$$

where *G* is the shear modulus of the tissue. It is possible to measure these quantities with well- defined experimental procedures, so that these formulas give an approach to model poroelastic expansions of the interstitial volume fraction. Moreover, the dilatation is potentially measurable by MR tagging methods, and hence the coefficient of proportionality between dilatation and pressure may be obtainable by a combination of MR imaging and simulation. Pending such advances, for simplicity, we have used a more restrictive assumption. Namely, we assume that both the fluid and solid constituents are separately incompressible. This is not a good assumption in the vicinity of blood vessels (e.g., for the region of parenchyma involved in perivascular flow), but, otherwise, it is reasonable with the very small pressures that pertain in CED. Under these circumstances, it may be shown that:

(12) e=δφ/(1−φ).

With this substitution, we get

(13) $$\delta \varphi =\left(\frac{\left(\alpha -\varphi \right)\left(1-\varphi \right)}{B}\right)\delta p.$$

This expression respects the limits of *φ*, namely that it cannot exceed α≤1 and is the one we have implemented. Moreover, it yields results similar to the previous expression (10) for most clinical measurements. In the incompressible limit, α→1, and 1−φ does not vary by more than a factor of two. On the other hand, in the limit of a suspension, B→0 and α→φ in which case the two expressions coincide. In this limit, the interstitial fraction increases directly with the increase in fluid content, and can do so without any pressure variation. In the future with better measurements of the poroelastic moduli, we can simply substitute an improved interstitial fraction–pressure relation.

**Figure 3:**
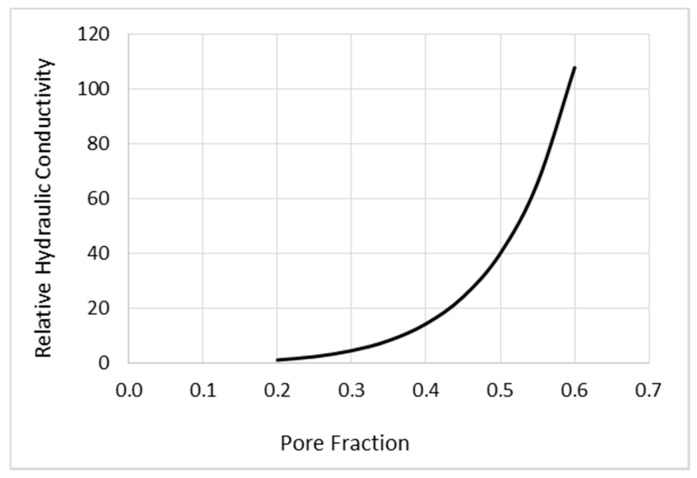
Relative increase of hydraulic conductivity, *K*, with increase in pore fraction, *φ*.

A nominal value of brain interstitial volume fraction, computed from space outside the cells and vasculature as a fraction of the total space, is often taken to be $\varphi_{0}=0.2$ [14] in both gray and white matter areas, with some variability. (This nominal value has been claimed to change rather dramatically between sleep (or anesthetized) and waking states [15,16].) Compared with a reference fluid conductivity *K* of 1 at this fraction, Figure 3 shows the variation of conductivity with φ that we have used in our work. It is of the form of the well-known Kozeny–Carman relations widely used in geophysics [17]. These relations have also been shown to be a good approximation for the behavior of fluid conductivity in tissue [18]. There are a number of variants of this formula, and we have used:

(14) $$K\left(\varphi \right)=K_{0}{\left(\varphi /\varphi_{0}\right)}^{3}{\left(\frac{1-\varphi_{0}}{1-\varphi }\right)}^{2}$$

where $K_{0}$ is the conductivity at the fiducial $\varphi_{0}$. However, we must remember that the geometry of the medium also changes with *φ* so that (14) really applies to an overall scale, while the tensor nature of *K* and its changes have to be separately taken into account. The literature on porous media uses the alternative term “pore fraction” for *φ*, and prefers to factor the conductivity thus: $K=\frac{k}{\eta }$, where *k* is called the hydraulic permeability and *η* is the viscosity. This has the advantage that the permeability describes the geometry of the medium (and has dimensions of area). However, since we do not delve into the mathematics of porous media, we continue to refer to *K*, noting that the viscosity might also alter with the interstitial fraction. Moreover, it is such a weak effect that we ignore it.

It is seen that the effect of increasing interstitial volume fraction is indeed dramatic. A threefold increase corresponds to more than two orders of magnitude increase in the conductivity. This is qualitatively (but not quantitatively) consistent with the dramatic increases reported in [11]. Increasing interstitial volume fraction is where the differences between most gray matter and white matter come into play. Most white matter regions are extremely expandable and thereby readily conduct fluid, as we have already shown from imaging results in the previous section, while gray matter shows little or no expansion or increase in conductivity.

**Remark** **1** (Anisotropy)**.** *We mention and give reference to a method of obtaining anisotropies of* *K* *from the diffusion tensor for water obtained by MRI. However, for our applications in this paper, we assume isotropy for* *K. This is because gray matter tends to be isotropic while white matter becomes so upon expansion, as noted. An exception is in the corpus callosum which has crossed fibers which do not reveal themselves in standard DTI: they require diffusion spectrum imaging, which does reveal further details of fiber tracts. We leave this as a task for the future*.

**Remark** **2** (Limits of white matter expansion)**.** *We mention, following Equation (7), that interstitial spaces are expandable without the need for increase in the pressure in the interstitium (e.g., Equation (5)) with δp=0: the resulting framework expands ( δe>0) if there is an increase in fluid content in a representative volume ( δφ>0). However, expanding interstitial spaces has limits, which we speculate is the cause for the “knee” observed in the pressure versus edema curve, namely the rapid increase of pressure and concomitant headaches in tumor patients past a certain level of edema, as when steroid or VEGF treatment is not used or has not restored the integrity of the BBB and reduced the edema. Given the side effects of such treatments, it is useful to be able to estimate when this knee occurs so that dangerous pressure rises are prevented. Suggestions for models for such effects are offered in [19].*

#### 2.1.2. Simulation Methods

From Equation (1), CED simulation requires several patient-specific fields to be computed: the diffusivity of the infused drug molecule in the tissue extracellular space; the velocity of extracellular fluid flow induced by the infusion, and average rate of loss of the drug molecule through capillaries, degradation, binding, etc. In addition, boundary conditions must be defined at the catheter infusion source and at the CSF boundaries which act as a sink for drug molecules.

Pressure and Fluid Velocity Simulation. Estimation of the fluid velocity field requires solution of the pair of equations described above (Equations (2) and (3)). These can be combined to generate a single differential equation that describes the relation between the pressure, the hydraulic conductivity of the tissue, and the capillary conductance, with boundary conditions for the pressure at the catheter source and the CSF.

**Figure 4:**
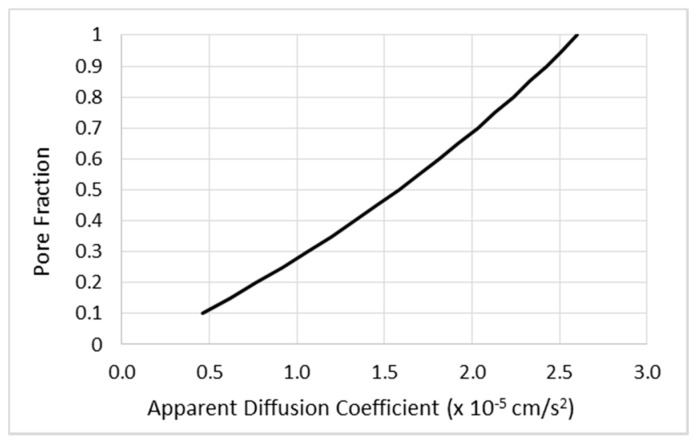
Estimation of pore fraction from measured apparent diffusion coefficient.

The hydraulic conductivity is estimated from the pore fraction as described in Equation (14). We estimate the pre-infusion pore fraction using the apparent diffusion coefficient (ADC) obtained from DTI. We use a slightly nonlinear numerical model relating ADC to pore fraction in which the ADC of normal brain (around 0.77 × 10^−5^ cm/s^2^) corresponds to pore fraction 0.2 and ADC above 2.6 × 10^−5^ cm/s^2^ is interpreted as CSF, pore fraction 1.0, as shown in Figure 4. As noted above, the pressure from the infusion alters the pore fraction and the hydraulic conductivity. In earlier work [1], the pore fraction in white matter was simply assumed to be elevated, with white matter regions identified using DTI. The procedure we have used to solve for the pressure in this work is iterative. We compute the fluid pressures for a given interstitial volume fraction *φ*. We then alter the fractions by the integrated form of the above equation, alter the hydraulic conductivities as previously described, and recompute the pressures. We find that the procedures converge adequately after 4–5 iterations. After solving for the pressure field, the velocity is obtained using Equation (2).

This approach to tissue expansion requires estimates of the Biot–Willis coefficient, *α*, and the drained bulk modulus, *B*, which are necessarily different in white and gray matter. We use estimated values for *α* and *B* in gray and white matter. Using DTI, we estimate the relative fractions of white and gray matter at each sample point, and linearly interpolate the local elasticity parameters using this fraction.

Capillary conductance, the product of capillary hydraulic conductivity, $L_{p}$, and the capillary density S/V (capillary surface area per unit volume) is taken to be uniform in unexpanded tissue. We take estimates of $L_{p}$ and S/V from the literature [20]. Tissue expansion should reduce the capillary density proportionally, and our simulation adjusts local S/V accordingly in each iteration.

Finally, the boundary conditions for the pressure simulation must be specified. We take the CSF to be the baseline pressure. In our simulations, CSF containing regions are identified and delineated from anatomical MRI and/or DTI images.

The catheter(s) provide the source pressure boundary. However, computation of the pressure along the catheter surface can be difficult, as most catheters do not provide a point source but rather a flow of infusate spreads along a portion of the catheter outer surface before penetrating into tissue. Backflow is a phenomenon that is part of the physics of the infusion process as shown in another seminal paper from the NIH group [21]. A-priori simulations of infusions require models or estimates of the pressure or velocity distributions as the fluid leaves the catheter in the presence of backflow. We analyzed this process and developed a model that could be used for predicting an approximate initial pressure distribution in the presence of backflow under the conditions of having an endport cylindrical catheter and no pre-stress upon insertion [22]. With complex catheter geometries such as stepped catheters, as well as potential damage to tissue upon insertion, such backflow models are fraught with uncertainties. We have developed a new method that can potentially be far more accurate, as indicated in Section 5. There is also some confusion in how people use the term backflow, which is often conflated with reflux along the catheter tract due to tearing of tissue. We confine use of this term to irreducible backflow along the outside of the catheter arising from the fact that the tissue framework is elastic. Further, people often measure backflow somewhat unsystematically: our approach to measuring it is described in [23].

In prior work, the pressure along the surface of the catheter was estimated based on catheter geometry and local tissue properties. Estimating local pressure is a tricky proposition and requires different models for different geometries. Mismatches between the source pressure model and the tissue simulation can give rise to errors in the fluid flux from the catheter source. For large infusions, small local variations in flow are less important than ensuring that the total flux matches the infusion rate. We therefore developed and implemented a new method to compute solutions to the equations for fluid flow with the boundary valued being a specified flux (related, via *K*, to the gradient of the pressure projected along a direction transverse to the boundary). This method is fully described in [24], where the “transverse” direction just mentioned is carefully defined. For isotropic *K*, this is the direction normal to the boundary. The paper just referenced also describes another application of the flux boundary value method that we expect will be helpful in reducing the unpredictability of the device-tissue interaction. This application is further discussed in Section 5 at the end of this paper.

*Concentration Simulation*. Local drug diffusivity within the extracellular space is estimated using diffusion tensor imaging. Based upon the DTI, we compute the diffusion tensor field by standard methods. This measure differs from the desired parameter in two respects: it describes the self-diffusion rate of water rather than drug molecules, and the measure combines the behaviors of intracellular and extracellular water. We estimate the extracellular drug diffusivity using a simple scaling relation based on the size of the molecule relative to water (see [1]). We assume the orientation of the tensor is the same for both measures. In any case, diffusion generally has a relatively small impact on CED distribution unless the drug remains in the extracellular space for a long time.

Losses of drug molecules through the various routes, if they can be modeled as exponentially decaying, can be combined and modeled with a single overall molecular loss rate. When modeling tracer molecules such at Magnevist, we generally ignore binding, internalization, and degradation as these are not significant factors over the duration of the infusion. In prior work, we have taken capillary loss to be uniform over all of the brain parenchyma. In normal brain tissue, this assumption is often adequate. However, tumors can have permeability that is vastly different from the surrounding brain tissue. We estimate the patient-specific permeability-surface area product in tumor using DCE. Our in-house software implementation employs the extended Kety model, described by the equation:

(15) $$C_{t}{(t)=v}_{p}C_{p}{(t)+K}^{trans}{[C}_{p}{(t)\;*e}^{-k_{ep}t}]$$

where “*” represents a convolution of the arterial input function, *C_p_*, with an exponential decay curve with time constant *k_ep_* = *K^trans^*/*v_e_*. *K^trans^* represents the volume transfer constant from plasma to extravascular extracellular space (EES), and *k_ep_* is the rate from EES back into plasma. Constants *v_e_* and *v_p_* are volume fractions of the EES and blood plasma, respectively. The plasma fraction is the extracellular portion of the total blood fraction, i.e., *v_p_* = (1 − Hct) *v_b_*, where Hct refers to Hematocrit, usually around 0.42. The goal is to estimate three independent parameters, *K^trans^*, *v_e_*, and *v_p_* such that, given an input function *C_p_*(*t*) that describes the concentration of the tracer in plasma in the region being evaluated over time, the model output most closely matches the observed concentration at each sample location. The input function is obtained by selecting a voxel in a large artery so that the entire signal is from the blood compartment, i.e., *v_b_* = 1.

If the tracer permeability is not too high (i.e., flux across the endothelium is limited by the permeability, rather than blood flow), then *K^trans^* is approximately equal to the capillary permeability-surface area product, the transfer rate for loss of infused tracer molecules from the extracellular space into the capillaries. If the simulation is predicting distribution of the Gd-DTPA tracer, we can use *K^trans^* directly as the capillary loss rate. Larger particles, such as ^124^I-HSA (and the protein-sized drugs used in the MR1-1 and D2C7 studies) will have much lower permeabilities. As it is not feasible to directly measure these rates in patients, we simply scale the Gd-DTPA permeability based on the molecule size, using values from the literature for permeability of differently-size tracer molecules as a guide. The measured small-molecule loss rate was divided by a factor of 200 for simulations of ^124^I-HSA distribution.

Finally, concentration boundary conditions must be specified at the catheter source and the CSF sinks. Using the backflow distance from the pressure computation, we assume that the catheter surface back to this point contains infusate at the infused concentration. Infusate that reaches the CSF is assumed to largely remain in CSF and not return to the parenchyma. Therefore, the CSF boundaries previously identified are used here as zero concentration boundaries.

### 2.2. Experimental Methods

The results obtained in this study were based upon imaging taken during two human clinical trials of immunotoxins, MR1-1 and D2C7, and in one case from a recombinant poliovirus infusion study. *Full information on these trials may be obtained at ClinicalTrials.gov under the Identifiers NCT01009866 for MR1-1, NCT02303678 for D2C7, and NCT1491893 for the recombinant poliovirus*. These may be consulted for all the Institutional approvals for the trials and for the imaging. We do not describe the clinical trials and results: see, for example, [25,26] for the MR1-1 and D2C7 studies, respectively and [27] or [28], for the poliovirus studies. Our purpose here is simply the evaluation of the infusion model against the data and conclusions, subject to more testing in the future, so that we may draw from the simulations the relative importance of the different variables in determining the distributions of the (therapeutic) particles (molecules). Our description of the study protocols is as brief as possible. The references given may be consulted for further details.

#### 2.2.1. Infusion Protocols

*All catheter placements in these studies were informed by the iPlanFlow™ simulations.* As mentioned, the algorithms and software simulating the fluid flow and drug distribution in BrainLab’s iPlanFlow™ were developed as described in prior work, and thus did not have the features introduced into the simulations used here. On the other hand, since they were placed based upon the features of a predictive planning procedure, the placements did benefit from image-guided planning.

The three MR1-1 patients reported here had four catheters placed in and around an enhancing tumor, each catheter being infused at a rate of 0.5 mL per hour. Chelated Gd-DTPA (Magnevist™ with Gd concentration of 1 mM), ^124^I-HSA (100 µCi per day of the PET nuclide ^124^I, a concentration of 2.08 μCi/mL), and the therapeutic molecule were co-infused. MR imaging was acquired after 2, 24, and 72 h of infusion while PET was acquired only at the 24- and 72-h time points. The infusion was discontinued during the imaging, with the PET imaging being done first, followed within 1–2 h by the MR imaging of the Gd tracer.

In the case of the D2C7 study, one or two catheters were placed in each patient, each catheter infused at 0.5 mL/h for 72 h. All infusions included the 1 mM Gd-DTPA MR tracer, with MRI performed only after 24 and 72 h. PET tracer (^124^I-HSA, 100 μCi total per day, concentration 8.33 or 4.17 μCi/mL depending on the number of catheters) was co-infused only in the first five patients, and PET imaging performed after 24 and 72 h of infusion.

For the recombinant poliovirus study, one catheter was placed into the enhancing tumor and infused at 0.5 mL/h for 6 h after an initial run of 30 min to expel 250 μL of air in the system. Magnevist MRI tracer was co-infused at 1 mM concentration and imaged after the infusion. No PET tracer was used in this study.

#### 2.2.2. Imaging Protocols

Since a particular improvement to the simulation is being able to simulate losses through the capillaries in the compromised BBB of the particular patient, it is essential that the pre-infusion imaging required for the simulation be carried out close to the time the infusions begin, to make sure that no tumor progression or change has occurred between imaging and infusion. This condition was always met in the studies reported upon here, since the lapse between pre-infusion imaging and infusion was at most a long weekend. The post-infusion imaging to measure concentrations of the reagents must also be made as close to the conclusion of the infusion as possible, so that the concentration measurements are accurate and can be compared with simulation, before substantial losses and degradation of the agent occurs. A three-Tesla Siemens Medical Systems “Trio” MR imaging/spectroscopy instrument (Siemens, Berlin-Munich, Germany) was used for brain diffusion tensor imaging. The PET imaging was performed using a GE Discovery 690 PET/CT system (GE, Boston, MA, USA). The PET scans were acquired along with a low-dose CT scan for purposes of image registration and attenuation correction.

MRI imaging for infusion measurement and simulation included sequences for T1 mapping, diffusion tensor field mapping, and tumor capillary loss rate measurement. For T1 mapping, a pair of 3D gradient recalled echo (GRE) sequences were acquired at flip angles of 6° and 34° with TR/TE = 20/4.92 ms, 256 × 256 matrix, 25-cm field of view (FOV), and 1-mm slice spacing. In the D2C7 study, B1^+^ field mapping images were also obtained and used to correct for B1 field inhomogeneities in the GRE images, using a pair of spin echo EPI acquisitions at 60° and 120° (TR/TE = 2500/16 ms, 128 × 128 matrix, 21.6-cm FOV, and 5-mm slice spacing. These images were acquired within a few days prior to infusion and repeated after 24 and 72 h of infusion. Additional anatomical imaging acquired at each of these time points included a T1-weighted MPRAGE (TR/TE/TI = 2120/4.1/1100 ms, 12° flip angle, 256 × 256 matrix, 25.6-cm FOV, 1.0-mm slice thickness) and 2D T2-weighted (TR/TE = 5000/100 ms) and FLAIR (TR/TE/TI = 9000/98/2500 ms) imaging with 256 × 256 matrix, 22–25-cm FOV, 3.0-mm slice thickness.

At the pre-infusion time point, additional imaging was acquired to obtain quantitative input for simulation. For diffusion tensor field mapping, a 12-direction, single-B0 2D EPI tensor sequence was acquired with three repetitions (b = 1000, TR = 8700–9100 ms, TE = 98–99 ms, 192 × 192 matrix, 23-cm FOV, and 3-mm slice thickness. Dynamic contrast imaging (DCE) was used to estimate the permeability-surface area product for simulations. An intravenous injection of 0.1 mmol/kg of gadolinium tracer (Magnevist) was tracked dynamically at 5-s intervals for at least 5 min using a T1-weighted 3D spoiled GRE sequence (T1 mapping with flip angles 3–35°, dynamic sequence with 3D SPGR imaging using 20 degree flip angle, TR/TE = 5/1.4 ms, 256 × 168 matrix, 25.1-cm FOV, and 5-mm slice thickness, 16 slices) centered around the tumor. Immediately following the DCE imaging, a post-contrast whole-brain T1-weighted 3D sequence was acquired (MR1-1 study: 3D SPGR, TR/TE = 19/4.92 ms, 25° flip angle, 256 × 256 matrix, 25.6-cm FOV, 1.5-mm slice thickness; D2C7 study: Fast 3D GRE (MPRAGE), as described above). The difference between pre- and post-contrast T1-weighted imaging was used to identify the enhancing region of the tumor.

#### 2.2.3. Concentration Measurement

The concentration of the Gd-DTPA tracer is estimated by first computing T1 on images obtained before and after the tracer infusion, and then estimating the tracer concentration from the change in T1. The variable nutation angle method [29] is used to estimate T1 from the 3D SPGR images with 6° and 34° flip angles. The pre- and post-infusion T1-maps are co-registered and differenced, and the change in T1 is then used to estimate Gd-DTPA concentration using the relation $\Delta \left(\frac{1}{T1}\right)=r_{1}C$, where r_1_ is the T1 relaxivity of Gd-DTPA at 3 T. We assume that the relaxivity does not change during the course of the infusion, that T2 effects can be ignored because of the low TE, and that any temperature effects are negligible.

The PET scans were attenuation corrected using the accompanying CT scan, producing a direct quantitative measure of ^124^I concentration. The paired PET/CT scans were electronically co-registered with the volumetric MRI scans using in-house software.

#### 2.2.4. Distribution Volume Measurement

Volumes of distribution are computed from the concentration maps using a threshold of 10% of the infused concentration. The number of concentration voxels above this threshold scaled by the voxel volume yields the V_d_ measurement. All infusions with Gd-DTPA employed 1 mM solutions, and thus a 0.1 mM threshold is required. The PET tracer levels are more complicated. The doses were calibrated to infuse a total of 100 µCi of ^124^I at the start of each day. MR1-1 patients received a total of 48 mL per day through four catheters, and thus about 2.08 µCi/mL. However, ^124^I has a half-life of 4.18 days, and therefore the dose after one day is reduced by a factor of 0.845 to about 1.76 µCi/mL. A fresh 100 µCi dose is prepared each day, and after three days of infusion the second day’s dose has decayed to 1.49 µCi and the first day’s dose to 1.25 µCi. Since this earliest dose is likely to by nearest the outer boundaries of the infusion, we use this concentration to compute the 72 h 10% threshold. The D2C7 patients in this study that had PET imaging had a single catheter infusing 12 mL per day, and so the ^124^I concentration levels are a factor of four larger, implying 10% thresholds of 7.04 µCi/mL at 24 h and 5.03 at 72 h. In a few cases, patients were imaged earlier or later than 72 h, and the thresholds have been adjusted accordingly in this analysis.

## 3. Results

We divide our reporting of the results in three subsections. In Section 3.1, we give a descriptive result primarily from one patient from the earlier MR1-1 study to illustrate several phenomena of interest. Following that, in Section 3.2, we summarize key features of distribution volumes from all of the studies in which both measured and simulated distributions could be obtained. Statistics on all of the infusions is available in Appendix A (Table A1).

### 3.1. Descriptive Results

**Figure 5:**
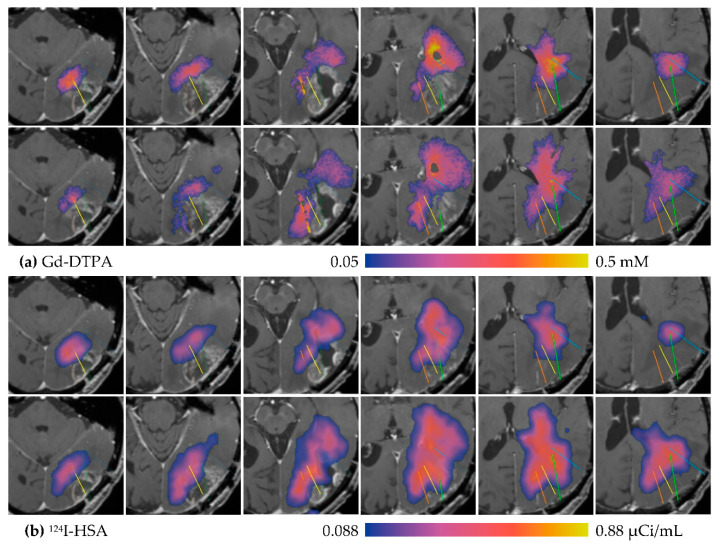
MR1-1 Patient 7 measured distribution (in color, overlaid on pre-infusion T1-weighted MRI, six axial slices, 10 mm apart). Colored lines indicate the approximate catheter placements, which are not entirely in-plane. The opaque portion of the line is inferior to the slice and the translucent portion is superior. (**a**) Measured Gd-DTPA concentration: (Top) after 24 h of infusion; and (Bottom) after 72 h of infusion. (**b**) Measured 124I-HSA concentration: (Top) after 24 h of infusion; and (Bottom) after 72 h of infusion.

We refer to MR1-1 Patient 7 for a detailed descriptive discussion. Three catheters were placed into the large enhancing tumor and one catheter placed a few millimeters posterior to the tumor, as illustrated in Figure 5. The top two rows (Figure 5a) show the distribution of Gd-DTPA after 24 h (top row) and 72 h (second row) of infusion. The concentration is displayed in color, overlaid on pre-infusion T1-weighted contrast MRI. The scale ranges 5–50% of the infused concentration. The bottom two rows (Figure 5b) show the same slices overlaid with measured ^124^I-HSA distribution at 24 and 72 h, using the same color scale at 5–50% of the infused ^124^I-HSA concentration after correcting for decay.

The distributions in this patient display several characteristics that are observed in most of the patients in this study. First, in comparing the 24- and 72-h time points, it is clear that the distribution volume has continued to grow in the final two days, but the rate of growth has slowed. The Gd-DTPA volume of distribution, V_d_, measured 50.1 mL after 24 h, and increased by only 22.1 mL over the next 48 h. After 24 h, ^124^I-HSA V_d_ measured 50.1 mL, increasing by 78.5 mL in the final 48 h. Second, compare the distribution of the small molecule gadolinium to that of the larger PET-labeled tracer. The spatial resolution of the PET imaging is coarser than the MRI, so these images tend to appear smoother and a bit larger than MRI-based concentration maps. Aside from this effect, the distributions of the two molecules appear to be similar, except in the region of enhancing tumor, where gadolinium distribution is largely absent (16.0% coverage at 72 h) while there is some coverage by HSA (54.3% coverage at 72 h).

**Figure 6:**
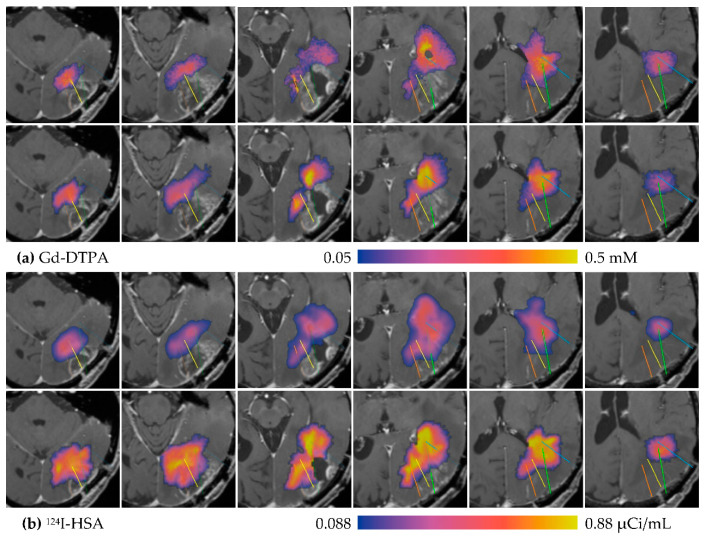
Comparison of measured concentration (top row) with the prediction from simulation (bottom row) after 24 h of infusion in MR1-1 Patient 7. Gd-DTPA distribution (in color, overlaid on pre-infusion T1-weighted MRI, six axial slices, 10 mm apart). (**a**) Measured (Top) vs. simulated (Bottom) Gd-DTPA concentration. (**b**) measured (Top) vs. simulated (Bottom) 124I-HSA concentration.

Simulations of the infusion in MR1-1 Patient 7 were performed, yielding predicted concentration maps for the MRI and PET tracers. These simulations are compared to the measured distributions at 24 h in Figure 6. The simulation of Gd-DTPA distribution in Figure 6a closely resembles the measured concentration in size, shape, and value. The ^124^I-HSA simulation in Figure 6b is similar in size and shape to the measurement, but the peak values are significantly larger. In this particular case, the *measured* relative concentration (fraction of infused) of the ^124^I appears to be a little less than that of the small molecule tracer, when it should be larger due to lower clearance rate. The simulation is more what we expect—shaped similar to the smaller tracer, but with higher concentration due to lower loss. The simulation for the small molecule is very much like the measured. We speculate (without further evidence, though) that the ^124^I is mis-calibrated.

**Figure 7:**
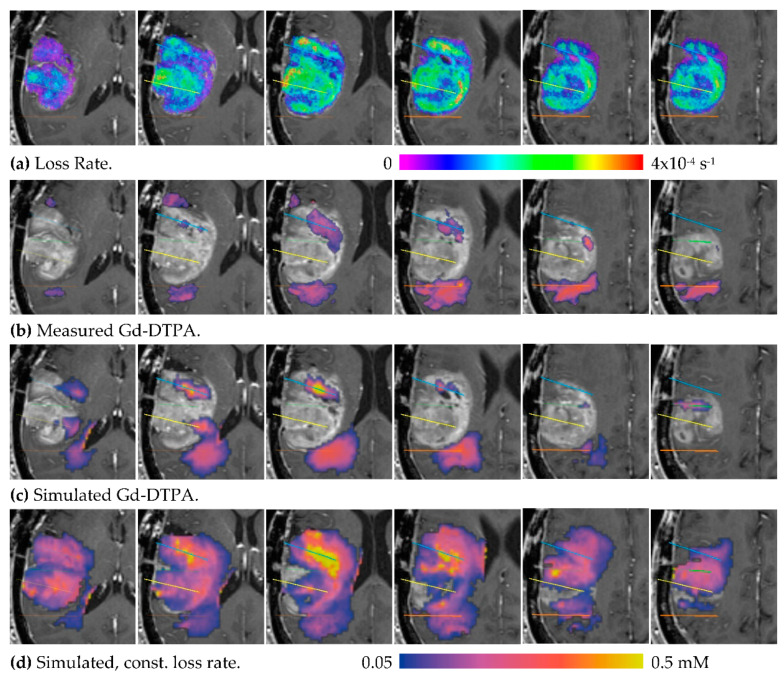
Infusion into large enhancing tumor (MR1-1 Patient 8). Loss rate and concentrations, in color, are overlaid on pre-infusion T1-weighted MRI, six axial slices, 6 mm apart. (**a**) Gd-DTPA capillary loss rate (technically, K^trans^) measured from DCE. (**b**) Measured Gd-DTPA concentration after 24 h. (**c**) Simulated Gd-DTPA concentration, using measured loss rate. (**d**) Simulated Gd-DTPA concentration, using constant normal-tissue loss rate (1 × 10^−6^ s^−1^).

Capillary loss can have a major impact on distribution. Consider MR1-1 Patient 8, where three catheters were placed into a large enhancing tumor and one placed just outside the posterior side of the tumor. The capillary loss rate for Gd-DTPA, measured prior to infusion using DCE imaging is shown in Figure 7a. The infused Gd-DTPA concentration measured after 24 h (Figure 7b) finds little tracer in the enhancing region. A simulation using the measured loss rate produced a similar result (Figure 7c). To evaluate the impact of the loss rate on the distribution, a simulation was run using the constant loss rate for normal tissue (10^−6^ s^−1^), as shown in Figure 7d. The simulation predicts that, without elevated capillary loss, the infusion would have covered most of the enhancing tumor. This implies that the infused fluid covers the tumor, but it is depleted of the tracer at a high rate through the capillaries.

**Figure 8:**
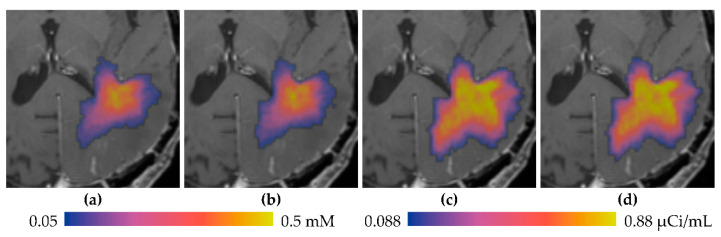
Diffusion has little impact on distribution. Simulations of Gd-DTPA and 124I-HSA concentrations with and without diffusion (72 h, MR1-1 Patient 7. (**a**) Simulated Gd with diffusion. (**b**) Simulated Gd without diffusion. (**c**) Simulated HSA with diffusion. (**d**) Simulated HSA without diffusion.

Diffusion has little impact on the distribution, at least where the concentrations are measurable. We may see this by simulation, which has the advantage over nature in that we can turn off diffusion. Figure 8 shows a simulation of both the small (Figure 8a,b) and the large (Figure 8c,d) tracer. Even after 72 h, and even for the small tracer molecule, the contours of the spread are not visibly enlarged.

One possible confounding issue in the simulation is the 250 μL of air in the dead space of the lines that is expelled at the start of the infusion. The air bubbles tend to stay near the catheter shaft for many hours. In this position, they may affect the backflow in ways that the simulation has no way to predict. In the three multi-day infusions (MR1-1), these mostly local effects are minimized by the large infusion size and did not appear to significantly impact the final distribution.

### 3.2. Statistics of Infusions and Simulations

*Key statistics of every imaging and simulation undertaken in these studies is provided in Appendix A*. In this section, the measured tracer distributions are presented and compared to the simulations.

#### 3.2.1. Total Distribution Volume

**Figure 9:**
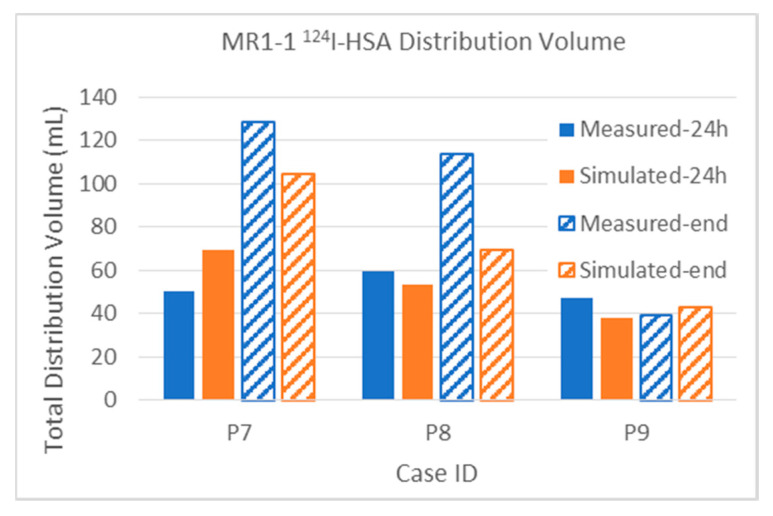
Comparison of measured and simulated ^124^I-HSA distribution in MR1-1 patients. Total infusion rate (from four catheters) was 2 mL/h. Patients 7 and 9 were infused for the full 72 h; Patient 8 was stopped early after 54 h of infusion.

**Figure 10:**
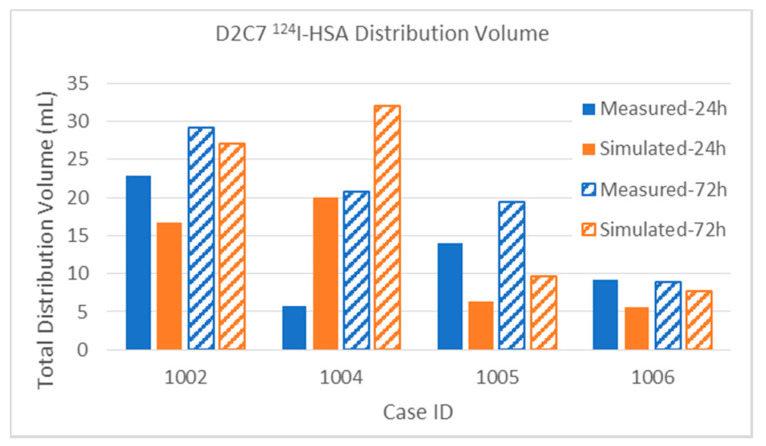
Comparison of measured and simulated ^124^I-labeled human serum albumin distribution in D2C7 patients. These four patients each had a single catheter infused at 0.5 mL/h.

The distribution of the albumin is expected to be closer to that of the therapeutic, because they are about the same size and shape, compared to the much smaller Gd-DTPA. Measured and simulated ^124^I-HSA distribution volumes from the MR1-1 and D2C7 patients are shown in Figure 9 and Figure 10, respectively. These MR1-1 patients each had four catheters placed and simultaneously infused at 0.5 mL/h. This resulted in a total of 48 mL at the end of 24 h, and 144 mL at the end of 72 h for Patients 7 and 9. The infusion in Patient 8 was stopped early, after 54 h, due to an adverse event, and therefore the patient received only 108 mL of total infusate. The agreement seems excellent except for Patient 8 at the end of the infusion. Further examination of the data revealed that the predicted backflow was at least 1 cm less than observed, and this observation introduced a further leakage path not accounted for in the simulation. There is a sulcus right alongside a catheter in Patient 8, and the PET shows a high concentration there that is directly centers on the sulcus. There also appears to be leakage to the subarachnoid CSF above another catheter. This is also the case where the diffusion is centered in thin bands, and so the low PET resolution has probably spread these narrow high-concentration areas into wider smoother regions.

Quantifiable PET imaging of ^124^I-HSA tracer was available for four of the D2C7 patients, as shown in Figure 10. These patients had only one catheter, infused at 0.5 mL/h, and thus the total infused volume was one-quarter that of the MR1-1: 12 mL at 24 h and 36 mL at 72 h. Patient 1002 could not be imaged until 67 h after the end of the (72-h) infusion, and some added diffusion (increasing the measured distribution) and some loss of tracer likely (decreasing the distribution) occurred in this time period. The simulation did not account for post-infusion changes due to loss or diffusion. Upon analyzing the images, the discrepancy in Patient 1004 was due to leakage of some of the infusate back into the CSF or subarachnoid space at the catheter entry point, while, for Patient 1005, the observed ^124^I-HSA distribution included spread through the adjacent cyst, and likely overestimation of distribution adjacent to the cyst.

**Figure 11:**
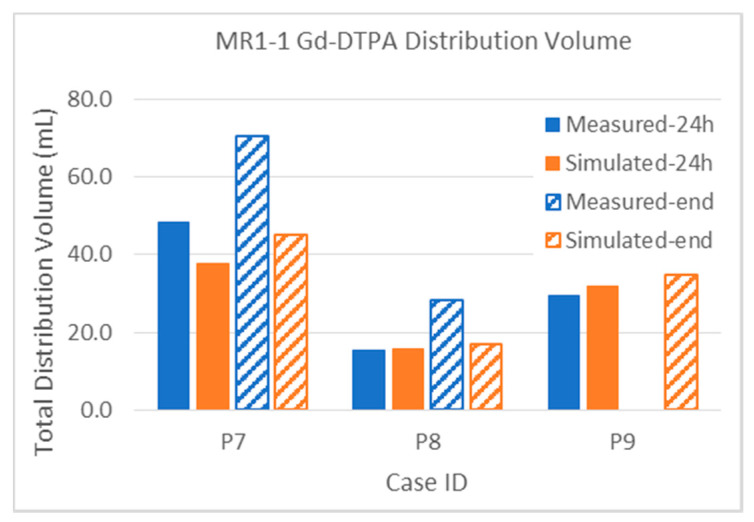
Comparison of measured and simulated Gd-DTPA distribution in MR1-1 patients. Total infusion rate (from four catheters) was 2 mL/h. Patients 7 and 9 were infused for the full 72 h; Patient 8 was stopped early after 54 h of infusion. No gadolinium was measured in Patient 9 at the 72 h time point.

Turning now to the small molecule MR contrast reagent Magnevist™, the MR1-1 results shown in Figure 11 show good overall agreement between the simulated and measured distribution volumes. The albumin distribution volumes (Figure 9) are significantly larger than that of the co-infused Gd-DTPA: about 70% larger in Patients 7 and 9, and over 300% in Patient 8. Nevertheless, the simulations clearly show the saturation effects and the losses, as discussed above. It may be argued that this is not directly relevant to the large biologic agent being infused: however, there are several clinical trials in which chemotherapeutics have been infused (e.g., [30,31]), and such results are relevant to these. For hydrophilic molecules, we may expect the loss rates to be similar to those of the Magnevist™, but, for lipophilic molecules, we will need to adjust the clearance rates.

**Figure 12:**
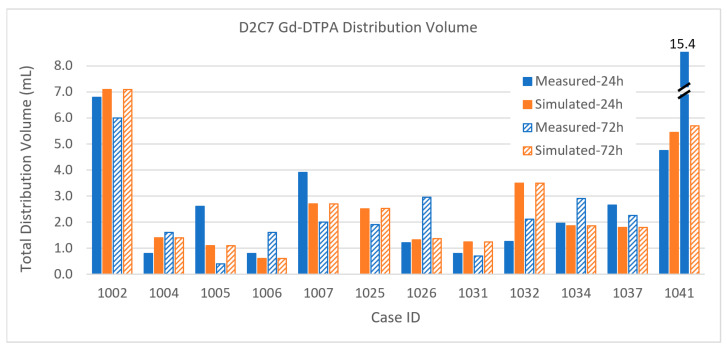
Comparison of measured and simulated Gd-DTPA in D2C7 patients. All of these patients had a single catheter infused at 0.5 mL/h. The Patient 1025 24-h measurement is omitted because the necessary MR imaging was not obtained. The Patient 1041 72-h bar shows a break because the value (15.4 mL) is beyond the scale of this chart.

The results for the small molecule in the smaller infusions in the D2C7 patients are shown in Figure 12. Again, remembering the extraordinary heterogeneity in the tumor tissue environment and the high loss rates for the small molecule, the results are in good agreement on the whole. We focus on the most discrepant results.

*Patient 1004*: The 24-h measurement is abnormally small that makes this one a poor match with the simulation, while the 72-h results match well. Perhaps there was more CSF leakage early than the simulation estimated.

*Patient 1005*: There is a huge reduction in the measured distribution from 24 to 72 h, not seen in the simulated distribution. It is possible that the measurement itself was in error, since there is no particular reason for this.

*Patient 1041*: The simulated distribution does not change much (from 4.74 to 15.41 mL) between the 24- and 72-h time points. On the other hand, the measured distribution markedly increased from 4.74 to 15.41 mL. We are unable to account for this particular discrepancy, although the simulation seems more believable and in line with other infusions.

**Figure 13:**
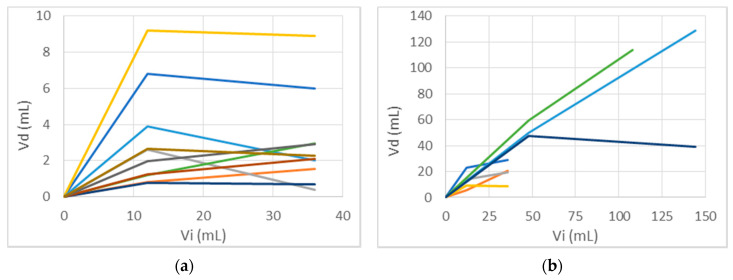
Diminishing returns. (**a**) Distribution volume vs. infusion volume of Gd-DTPA in D2C7 patients who had one catheter. (**b**) V_d_ vs. V_i_ of ^124^I-HSA in MR1-1 patients and D2C7 patients with quantitative PET imaging.

In the distribution measurements above, it can be seen that, although the volumes usually increase between the 24- and 72-h measurements, the rate of increase (V_d_/V_i_) is often much lower than in the first 24 h. This is most pronounced for the small molecule Gd-DTPA. In Figure 13a, V_d_ vs. V_i_ is plotted for the Gd distributions in D2C7 patients that had one catheter (and thus 36 mL total infusion volume). We have selected the single catheter infusions because they are a large enough set that have the same Vi so they can be compared well on the chart.

In all of these cases, there was a reduction in the slope, V_d_/V_i_ after 24 h. Note that in some cases there is a reduction in measured Gd distribution at 72 h. This may indicate that the distribution size has plateaued, and the high clearance rate makes this measurement sensitive to the amount of time between the end of the infusion and the scan.

Figure 13b plots the ^124^I-HSA distributions of all of the MR1-1 and D2C7 patients for which there is quantitative PET data in this study. Four showed a sharp decrease in slope, and three maintained a relatively steady rate. Note that even for this large HSA molecule, and even for the first 24 h, the ratios Vd/Vi usually are less than 1, instead of being closer to 5 as would be expected at the canonical interstitial volume fraction of 0.2. Further details on the distribution volumes are discussed, and are fully reported in Table A1 (Appendix A).

#### 3.2.2. Tumor Distribution Volume

In this section, we examine the measured and simulated distributions within the enhancing tumor, which were identified by differencing pre- and post-contrast T1-weighted MRI from the pre-infusion scans. All of the patients described had regions of enhancing tumor that were targeted with one or more catheters. Coverage of these regions by the infusate is of particular importance, and also can be the most difficult for computer modeling, as their properties can widely vary and are quite different from normal tissue.

**Figure 14:**
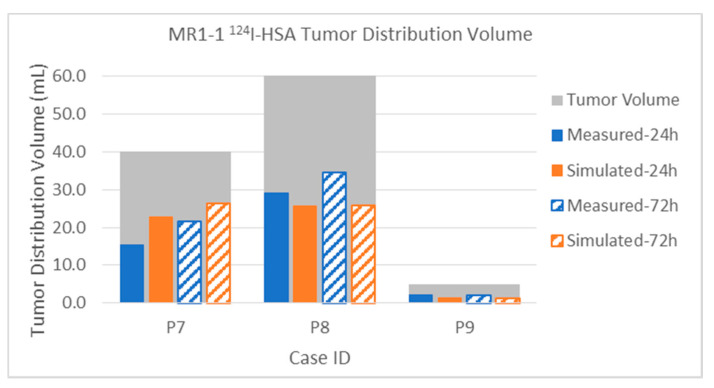
Comparison of measured and simulated ^124^I-HSA distribution within contrast-enhancing tumor in MR1-1 patients. Gray bars show tumor volume.

The intra-tumoral distribution volumes of the ^124^I-HSA in MR1-1 patients are plotted in Figure 14. For comparison, the volume of the enhancing tumor region is show as a gray bar behind the tracer data, to allow visualization of the relative size and coverage of each tumor. The data show excellent agreement between simulations and measurement, even for the very small tumor in Patient 9. The infusion coverage of the tumor is also reasonably good, although not complete.

**Figure 15:**
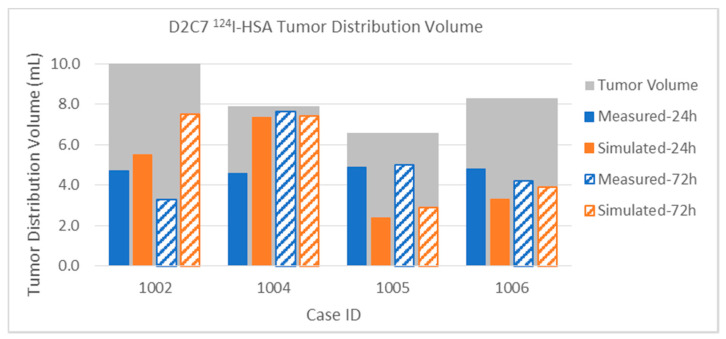
Comparison of measured and simulated ^124^I-HSA distribution within contrast-enhancing tumor in D2C7 patients. Gray bars show tumor volume.

The distribution of albumin in D2C7 patients is shown in Figure 15. As noted above, the end of infusion (72-h) PET scanning on Patient 1002 was delayed by 67 h, and at that time showed a lower distribution in the tumor than the 24-h measurement. Simulation, which did not attempt to account for this 67-h delay, showed increased coverage at the 72-h time point. This suggests that there was significant capillary loss of the large molecule tracer within the tumor over the 67 h. Recall that the total distribution volume (Figure 10 and attendant discussion) had the opposite effect, possibly because the total distribution is dominated by the spread in the tissue with intact BBB.

The PET tracer measurement in Patient 1004 showed nearly complete coverage of the enhancing tumor from the single catheter placement (58% at 24 h, 97% at 72 h). Some loss of infusate was observed due to backflow along the catheter into the subarachnoid. Simulation predicted similar distributions of albumin to those observed, but growing faster than measured, which may be due to the backflow loss observed. Lower than measured distribution size by the simulation in Patient 1005 appears to be related to cystic or necrotic regions of the tumor that are not enhancing. The infusate tends to distribute widely in such fluid-filled regions, and due to the limited resolution of PET imaging, signal can sometimes blur over into adjacent enhancing regions that are not actually covered by infusate, leading to overestimation.

Finally, the Gd-DTPA distributions within tumor are described below. Due to the elevated capillary loss rates of small molecules in enhancing tumor, Gd-DTPA tumor coverage is much lower than that of albumin for both sets of patients. This high loss rate tends to be the major determinant in distribution within the tumor, and it is thus important to obtain an accurate capillary loss rate estimation for the simulation. The gadolinium loss rates are so high in enhancing regions that the time between disconnecting the patient from the pump and performing the MRI scans (up to 2 h) can affect the measurement. The implications for either attempting contrast-enhancing tumor coverage with small molecules that easily cross the blood–brain barrier, or for using such molecules as tracers, are discussed below.

**Figure 16:**
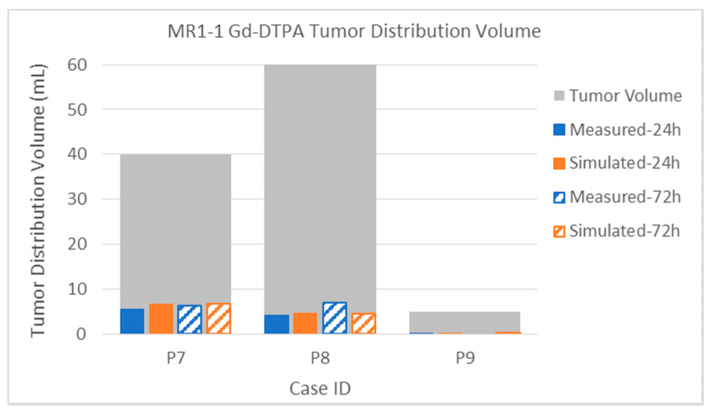
Comparison of measured and simulated Gd-DTPA distribution within contrast-enhancing tumor in MR1-1 patients. Gray bars show tumor volume.

The intra-tumoral gadolinium distribution in the MR1-1 patients is shown in Figure 16. The measured distributions in tumor are much smaller than the co-infused albumin. Nevertheless, the simulated distribution volumes are close the measurements. Recall that, in Patient 9, no gadolinium was measured at the 72-h time point. The distribution in tumor is very small (0.5 mL) at the 24-h time point, but the simulated very similar (0.6 mL).

**Figure 17:**
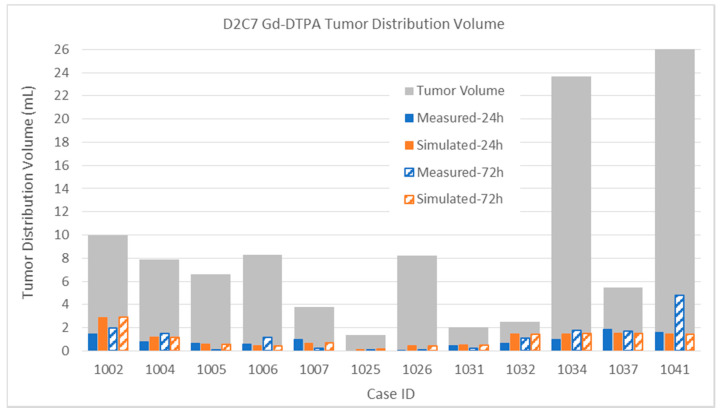
Comparison of measured and simulated Gd-DTPA distribution within contrast-enhancing tumor in D2C7 patients. Gray bars show tumor volume.

The intra-tumoral Gd-DTPA distributions in the D2C7 patient are shown in Figure 17. With only one catheter, the intra-tumoral distributions of the small molecule tracer are much smaller than those of the MR1-1 patients. As in the MR1-1 patients, the gadolinium coverage of the tumors was typically 3–8 times smaller than that of the measured albumin in the patients that had PET imaging. The intra-tumoral gadolinium volumes are sometimes smaller in the 72-h measurement than the 24-h one, due to variations in the time delay for obtaining the MRI scans, as described above. Simulated distributions largely correlate with the measurements, within the margin of error due to this delay (combined with high loss rate). Gadolinium distribution in Patient 1041 was atypical, in that the measured distribution volume increased by a factor of three between 24 and 72 h. In most other patients, there was little increase in intra-tumoral Gd-DTPA after 24 h.

#### 3.2.3. Error Measure

We may summarize a measure for the error in the simulations of the distribution volume by a percentage of the absolute values of the errors, relative to the measured distribution volume, and weighted by the latter. We explain the definitions of the terms used in Table 2 below.

All percentage errors are shown to the nearest significant digit only. The bias is the sum of the signed individual errors (i.e., the difference between each simulated and measured distribution volume), divided by the total measured volume and expressed as a percentage. The L1 error, on the other hand, is the sum of the absolute values of the difference between measured and simulation volumes for each case, divided by the total measured distribution volume, and expressed as a percentage. We have computed the standard deviation the data comprising the individual bias errors, and these are numbers smaller than the L1 errors, but it is not clear what they might mean. It should be noted (see Table A1 in Appendix A) that there is an enormous range of two orders in magnitude in the volumes so that, particularly for the small volumes, a small deviation in the prediction can be a large fractional error. Further, there are large losses of the infusate molecules, both through the capillaries of the tumor and from the parenchyma into the CSF. The ratio of the distribution volumes to infusion volumes is seldom greater than one, and frequently far less than that. Given a nominal interstitial volume fraction of 1/5, we would expect instead a distribution volume several times that of the infusion volume. Indeed, for small volume infusions in intact brain, this is what we can see (as, for example, seen in the graphs shown in [32]). In large infusions as studied here, there is ample opportunity for infusions to find paths leading out of the brain, be it across the capillaries or into the sulcal and other CSF spaces. Despite all this (and see below on the limitations of the anatomic imaging), we have been able to take into account such losses to produce the level of agreement shown in detail in the images and in summary in the charts and tables. The bias for the distribution within tumor is small in all cases compared with that for the total volume. This, we believe, reflects some of the uncertainties in accounting or not for distribution within CSF spaces.

## 4. Discussion

We divide our discussion into three parts: the first a reminder on the quantitative results presented above, the second on “lessons learned” that leans heavily on the descriptive results presented in Section 3.1, and the third on phenomena we have omitted in our simulations, and the potential consequences. We defer future research directions to the Section 5.

### 4.1. Quantitative Results

We discuss our quantitative results in detail in the previous section where they are presented. We therefore do not repeat that discussion here, but restate the principal conclusions. (1) An encouraging feature of the simulations is that, despite the more than two orders of magnitude in the variation of distribution volumes (the smallest is about 0.5 mL, and the largest about 150 mL), the simulations do track the variations and clearly differentiate between the good and the bad infusions, and between rapidly clearing and long residence time molecules. (2) The detailed results often do not match but there are a number of reasons for this, some of which are just a matter of accounting differently in the measurement versus the simulation. (3) However, some of these discrepancies are due to factors beyond the ability of the simulations to predict. These include large bubbles, or tearing of tissue upon catheter insertion, for example. Such phenomena are part of “original sin” in the infusion procedure. The best way to overcome these limitations is, we believe, with early real-time monitoring, discussed below in Section 5, under *Unpredictability*.

### 4.2. Qualitative Conclusions

We use the categories mentioned in the Introduction, namely those of the response of the interstitium, the behavior of the agent therein, and the resulting unpredictability of the infusions if based on intuitive considerations of standard clinical imaging.

- *Anisotropic distribution arises from expansion of the intercellular space and corresponding increase in hydraulic conductivity rather than from cell geometry and directionality of the brain pathways*.

We show above that pre- and post-infusion FLAIR indicate increased water fraction co-located with infusate tracer, which demonstrates that expansion occurs (Figure 2). Pre- and post-infusion DTI show increased diffusivity co-located with diffusion tracer, which suggests that expansion leads to increased conductivity; moreover, this edematous white matter displays low fractional anisotropy (Figure 3). This shows that directionality is not required to obtain white matter preference. A mathematical model showing the extreme sensitivity of conductivity to interstitial volume increases (Figure 5) is both consistent with other studies and is required to agree with the data presented here. The simulation comparing the effects of expansion and anisotropy as potential causes of white matter preference show further credence for this assertion.

- Pre-infusion DTI does not directly predict distribution anisotropy.

The dynamic response of the interstitium to the infusion makes this obvious, as discussed in detail in Section 2. A poroelastic model is key in understanding and estimating the responses.

- Advection is size-independent, over a range of sizes from small molecules to large proteins and beyond.

In particular, we show this via simulation in previous sections, where the distribution of the small molecule Gd (~700 Da), with no allowance made for clearance, is shown comparable to that of the large molecule albumin (~66 kDa). Further, in previously published work in porcine brain, we have shown this size independence by direct measurement (Figure 6 of [32]), which clearly displayed essentially the same distribution volume for a Gd-labeled albumin tracer as for a large Gd-based nanoparticle tracer of size 30 nm, which is larger than an adeno-associated viral delivery agent. These tracers do not bind, and the infusions were in intact pig brain (so that capillary losses did not occur at least during the time of the infusion). Thus, these were measures essentially of advection without any confounding factors and showed the same distribution so that a “retardation factor” (see Introduction) was not called for, even for the nanoparticle! (Other researchers have shown benefits from pre-expanding the interstitium for large particles such as viruses [33].) We may conclude that molecular size does not significantly impact distribution due to advection. However, before we conclude that tracer size need not be matched to the drug size to achieve a good estimate of distribution, we must ascertain that the tracer concentration is large enough to remain measurable relative to the local loss rate. We further discuss these points below.

- Capillary losses result in widely varying residence times in tissue.

In normal tissue, loss rates are measurably different for different-size molecules. Loss rates can be orders of magnitude larger in active tumors, as shown in the previous section in comparing chelated gadolinium and I-123-labeled albumin concentrations. Thus, *tracer compounds will not appear to have the same concentrations as drug molecules with different size/permeability, especially near active tumor*. This is an unsurprising result, and small tracer molecules are likely to underestimate distribution especially near active tumor, because their concentrations may fall too low to be detected. The chief difference between the spread of a small molecule (MR contrast reagent Gadolinium chelated in a small molecule) and a large protein (albumin) is not the advection which is essentially the same for both but that the former is rapidly removed from the tissue by efflux through the vasculature, particularly in tumors that have compromised the BBB. We get excellent agreement in simulations with the experimentally observed distributions by accounting for this loss quantitatively via DCE imaging.

- Diffusion effects are not a significant part of CED infusion distribution for “biologics” or protein-sized molecules.

We discuss this in relation to Figure 8 in the Section 3.1 on descriptive results above. It is actually remarkable that, despite the very small fluid speeds that pertain at the boundary of the distributions, convection can remain dominant over diffusion at least for concentrations above the threshold we have chosen (10% of infused). See also the next point.

- *Saturation of distribution volume*.

We show the concentration within tissue as a function of time in Figure 12. The saturation effects on the small molecule (Figure 13a) are explainable (by simulation in particular) as due to losses across the capillaries as well as entry into the CSF spaces. In addition, the spread is slowed due to the considerable reduction in fluid speeds as we move away from the infusion port. Despite this, it is remarkable that convection continues to be effective as seen in a couple of the cases shown in Figure 13b for the large molecule, absent leakage into CSF spaces.

### 4.3. Phenomena Omitted

- *Perivascular and glymphatic spaces*

In the simulations described above, we have not accounted for the endogenous flows in the perivascular and glymphatic spaces which have received considerable attention in the last decade. (Figure 3 in [34] provides a conceptual sketch of these various pathways.) The traditional interstitium is the space outside cells and outside blood vessels usually treated in a relatively undifferentiated way. However, it is claimed that the space surrounding blood vessels is a region of considerably enhanced fluid conductivity. A third set of pathways are internal to glial cells, hence entirely outside of the traditional interstitium. However, such pathways are primarily fluid pathways and do not concern therapeutic solutes. We first make some estimates of these effects, and then discuss the data we have. The estimates provided by the researchers suggest flow speeds of the order of a micron/second [35]. These are quite substantial flow speeds. In fact, a simple geometric calculation, as well as direct measurements [24,36], shows that flow speeds due to the infusions studied in this paper as close as 1 cm from the catheter are less than micron/second. One might expect such flow speeds to seriously affect the fluid and particle distributions, and that any simulation which neglected these would be more substantially in disagreement with data than ours seem to be. There are two reasons this does not seem to be the case.

Let us first consider the case of such speeds occurring near the smaller capillaries which branch every few microns or so. In such a case, the putative convective speed becomes effectively diffusive due to repeated branches. This process is well known as hydrodynamic dispersion, ([17], Chapter 10) and the effective diffusivity is D ~ LV where L is the mean length between branchings and V is the convective speed. The result is an added diffusivity of the molecule of the order of 1/100th μm^2^/ms which is even smaller than the intrinsic diffusivity of the albumin-sized molecules we have been studying and which, as we have seen, plays little role in the distribution. Such an effect would not be measurable in the presence of the much larger advective (convective, as it is usually called) speeds of the CED.

The second case is that of the larger blood vessels which have lengths of centimeters and more. Unfortunately in our examples, all such vessels are superficial (outside the parenchyma where the infusions are planned) and any perivascular spaces are far from the regions of convective spread, such as the edematous white matter shown in Figure 1 and Figure 2. Thus, again, we have no experimental authority to include any such endogenous flow. As stated in the Introduction, these pathways are the subject of much controversy at present, so we aim to be guided by experimental necessity, which we have so far not been able to see. Nevertheless, we caution that we are dealing here with large infusions. Even if perivascular spaces were discernible for small infusions, they merge into the larger interstitium very quickly. We have shown this in primate studies in other work (see discussion on perivascular loss in [37]). In general, all such pathways fall under the rubric of local alterations of the hydraulic conductivity which the model and software can certainly accommodate, once the requisite information is available.

As for the glymphatics, they would affect the term that encapsulates the sinks and sources of fluid in Equation (3), and we can incorporate them as results demand, or information on their ultrastructure becomes available.

- The tumor interstitium

Edema is a well-known consequence of malignant tumors [38], and there are detailed studies and models of elevated interstitial pressures in tumors [39,40,41,42]. Such elevated pressure also drives interstitial flow, as well as edema due to solutes such as albumin now entering interstitium from the blood vessels across the compromised BBB. However, it should be noted that this pressure-driven flow has quite different characteristics from that due to a pump with a fixed flow rate. In the latter case, the absolute level of the fluid conductivity makes no difference to the spread of the fluid (though differential region conductivities do). The pressure simply adjusts according to the conductivity. In pressure-driven flow, it is the flow that adjusts to the conductivity; a large conductivity means more flow. We have made a detailed modeling study of the consequences of this [43], and shown quite substantive effects due to moderate elevations of tumor interstitial pressure such as posited in the literature.

Nevertheless, no accommodation for elevated tumor pressures has been made in our simulations. We have attempted to obtain pressures from the monitors used in these infusions, but they have been so variable and erratic that we failed to discern if there was any elevated pressure due to tumors in these patients or not. It must be borne in mind that the patients are subjected to fairly aggressive supportive steroid treatment which may result in closing of the BBB so that elevated tumor pressures are not obtained in the cases we studied, but all this remains speculative. All we can say is that our simulations without accounting for elevated tumor pressures seem to account for all the principal features, both quantitative and qualitative (with the caveats mentioned in the previous section on loss of infusate) of the infusions. Another speculative reason we may suggest is that our model for increased conductivity Equation (14) subsumes some of the effects mentioned in this and the previous bulleted item. Clearly, the comments in this and the previous bulleted item require future research to either support or dispose of our speculations.

- *Binding of drug molecules*.

Binding may or may not affect the free drug concentration depending on a highly nonlinear “binding site barrier” phenomenon arising from binding kinetics. We provide a detailed discussion of this phenomenon, along with prior misunderstandings of its mechanisms, elsewhere [12] (see Figure 3 in that paper and attendant discussion in the text). The effect of strong binding has been known as the “binding site barrier”, and it limits the free drug concentration available beyond the first layer of cells available for binding. In the pioneering CED modeling paper [4], this effect is derived as a linear term in the concentration in an equation such as (1) above: however, we show [12] that this is quite nonlinear. When the infused concentration is even *2* *× receptor concentration*, there is only a small effect on the time to reach a destination with interstitial concentration close to that infused.

## 5. Conclusions

We summarize our conclusions from the discussion above and suggest further future directions to address present shortcomings.

**Figure 18:**
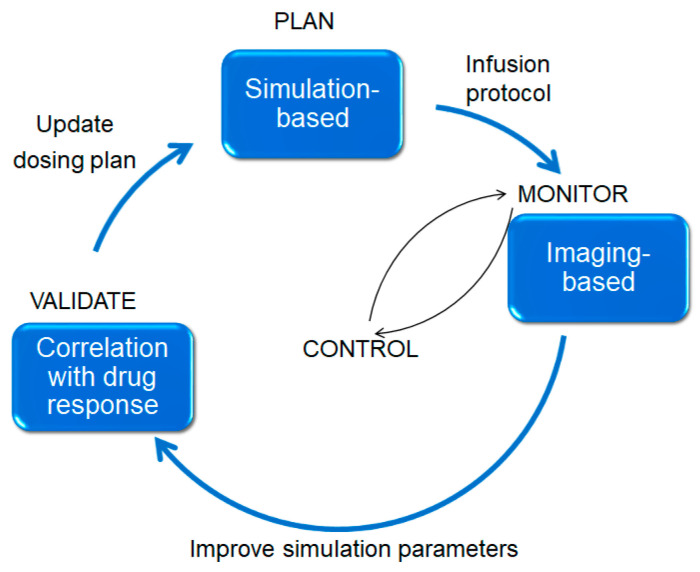
Interventional feedback loop.

- *Interstitium responses to infusion of fluid*: Simulations and planning can reduce variability. Figure 18 illustrates an approach we hope to pursue and test in the future. An a priori plan (top) is used, in conjunction with neurosurgical expertise, to position the catheters to obtain best coverage of the target region. The infusion proceeds and a surrogate tracer is imaged (if conjugating the actual therapeutic particle with an imaging reagent is not possible).
- *Losses and binding*: Compromised BBB has significant effects on small molecules, so that their distribution is quite different from that of larger particles in areas of high BBB permeability. *The principal uncertainty in quantification of the DCE is the arterial input function, as has been noted many times in the literature* (see also Section 5.1.2). To quantify the losses of a range of small chemotherapeutic reagents, such as being used in other CED studies currently (see, e.g., the reviews [30,31]), more pharmacokinetic studies will be needed, as proposed in [44,45]. However, for larger biological agents, it appears these losses are not so significant that we are unable to obtain reasonably accurate simulations. We discuss binding effects above.
- *Unpredictability of device-tissue interaction:* We show several examples of unpredictability where we discuss our results (Section 3). The device–tissue interaction, as well as the effects of catheter geometry, are hard to model. Backflow and bubbles are both unpredictable and have large impacts on the subsequent distributions. As noted, our backflow model was an approximation to only one geometry of catheter—the cylindrical endport—and did not account for pre-stress. Both of these have very significant effects on backflow (see, for example, [32,46]). In addition, porous catheters, deemed useful for delivery over a large area with advantages of bridging over sinks [47], require a quite different model for outflow from the catheter [48]. However, in these large infusions in human brain, the effects matter less as long as the infusion does not start close to CSF spaces. It would be more robust to measure the flux at the start of the infusions for distribution estimates. We have developed and tested these ideas in animal models: the experiments have been reported in [36,49,50]; and the theory behind it as well as some of the results have appeared in [24]. The protocol for the studies reported here did not allow for application of these ideas, as discussed in Section 2.2.2, nor were the methods sufficiently developed at the time of these trials for implementation. When implemented, this would likely avoid the unpredictability arising from having to model either the specific catheter or its backflow characteristics. We mention above that priming and other methods can reduce or nearly eliminate large bubbles, but a more practicable method needs to be found. Thus, we conclude that backflow is device and device–tissue interaction-dependent and unpredictable, and it can have a large impact on the subsequent distribution. If the infusion is not close to the CSF, this tends to matter less as infusions become larger. However, backflow and device–tissue interaction will matter in small infusions and can be incorporated into the planning system by the real-time MR methods referenced above.

### 5.1. Future Studies

Some future developments that may prove valuable are discussed below, grouped as belonging to modeling and simulations, to imaging, and to delivery devices, respectively. There are many other developments that would be valuable for better therapeutic outcomes, but these are beyond the scope of this paper.

#### 5.1.1. Modeling and Simulations

We list a few additions to the capabilities of the algorithms that would be useful to develop in the near future.

*Direct simulation of fluid flux.* Currently, the fluid pressure is simulated, and then a numerical difference procedure is performed to obtain the pressure gradient, and therefrom, via Darcy’s law, the fluid velocity. It may be numerically more robust and stable to directly simulate the velocities. We have developed the mathematical approach in unpublished work, but all implementation remains in the future.

*Inverse planning.* The current simulation computes the fluid and drug distribution based on catheter placement and flow protocol. There is a need for automated or semi-automated catheter position optimization into regions of the brain designated for coverage by drug or therapy. It would be beneficial to have a simulation accept input of target infusion coverage volume, as well as input of preselected compact regions for catheter placement (or several preselected putative catheter placements). The simulation could run from the set of points selected or a set of points which “triangulate” the region. Then, based on the percentage of target volume covered, an optimal placement of catheters for infusion could be the output. This is a partial and interactive solution to the full inverse problem, but one more likely to succeed than a more automated approach. The quality of the solution will, however, depend on the quality of the manual choice of initial points and region.

*Endogenous sources of flow and pressure.* The equations whose solutions have been incorporated into software (see PMB1) do not account for endogenous interstitial flow. Such flow in normal brains is not significant compared to that from high flow rate infusions, but it may be significant in either chronic infusions or, more pertinently, in brains with pathological states of disrupted blood–brain barrier (BBB), where there are endogenous sources of fluid as well as serum proteins from the vasculature that alter the oncotic pressure in the interstitium [43]. It would be useful to modify the algorithms to account for such endogenous sources, whether they may be either pressure or flow driven.

*Incorporation of other catheters and protocols.* Ramped and other protocols for flow rate control should be allowed for, in addition to the current single flow rate input required by the simulation.

*Incorporation of other pathways and transport phenomena*.

As discussed in the text, we have not incorporated the perivascular and glymphatic pathways, both because the imaging does not allow us to delineate the higher hydraulic conductivities in such regions and because the data we have encountered in these large volume infusion studies do not seem to demand it. As for the transport of the therapeutic particles, we have not for example incorporated any axonal transport processes that have been postulated for viral vectors. We would need both the appropriate fiber tracking imaging as well as experimental data on such transport for a useful simulation of such phenomena.

Finally, we mention that a good model for fluid and particle transport in the brain can have a myriad of applications in research and in clinically useful applications. Some potential opportunities are listed in Table A2 (Appendix B).

#### 5.1.2. Imaging

We discuss above the principal sources of uncertainty in the simulations: delineating the CSF spaces, quantitating the capillary loss rates, accounting properly for the poroelasticity, and predicting the effects of the device and insertion on the initial backflow and leakage pathways being among these. There are several advances in imaging (aside from the real-time imaging updates already discussed) which, if feasible, would allow improved predictions for planning delivery. For example, if we could directly measure the interstitial fraction upon infusion, this would obviate much uncertainty in the calculation of the pressure and velocity. There is a lot of uncertainty in the algorithm around the time-varying pore fraction. A key two-step process is used to compute likely hydraulic conductivity from assumptions on the pore fraction. A periodic imaging method that updates the pore fraction map would remove a lot of uncertainty in the algorithm. We would not need to rely on the approximations to poroelasticity made in the calculation or on the estimates of uncertain poroelastic parameters.

If it is too cumbersome to arrive at the pore fraction, poroelastic imaging would be of help so we can measure the parameters in vivo. Better delineation of the components (fluid and tissue classified according to its poroelasticity and white matter content) may be addressed either by higher resolution imaging or by more sophisticated methods such as multicomponent T2 analysis, which has a large and growing literature and would allow us to derive a CSF component per voxel to better determine when a small sulcus was in a voxel. New MRI methods to better characterize tissue in hopes of better predicting changes in hydraulic conductivity would be most welcome.

#### 5.1.3. Delivery, Devices, and Device-Tissue Interaction

There are many catheters now available which are significantly more sophisticated than the ones used in this study. It is beyond the scope of this paper to refer to such devices, which are now being used extensively in newer CED studies. We point out that, to ensure adequate delivery into the margins, catheters such as the Cleveland Multiport Catheter are now being used. It would also be advantageous to explore an inside-out approach, as explored in [51], with some suggested by one of us (see Figure 4 in [12]), with some further preliminary work based on a design in [52].

To conclude, there is much room in the technology of delivery, imaging, and simulations for further improvement of the reliability and reproducibility of infusions of appropriate therapies for brain cancer, and other serious diseases of the central nervous system, as well as solid tissue cancers in the rest of the body.

## Figures and Tables

**Table 1:** Glossary of Selected Symbols.

Symbol	Stands For
*p*	Fluid pressure in the interstitium; function of position (tissue place) but not time
v	Darcy velocity in tissue (a vector, or more precisely, a covector); function of position
*c*	The interstitial concentration of a free or unbound particle (therapeutic or marker molecule); function of position and time
*b*	The interstitial concentration of the bound particle (therapeutic); as for c
*K*	The hydraulic conductivity in tissue (a second rank symmetric positive definite tensor, which is a matrix in a specific coordinate system); can depend on position
D	The diffusion tensor of the particle in tissue (a second rank symmetric positive definite tensor, which is a matrix in a specific coordinate system); as for D
φ	The interstitial volume fraction, also known as the pore fraction particularly in the geophysical literature of porous media. Depends on position (tissue type)
α	In Section 2.1.1 (porous medium physics related) poroelastic coefficient known as the Biot–Willis parameter; a constant
β	(The square root of) capillary conductance; depends on position according to BBB compromise
*B*	Generally, a bulk modulus of elasticity; specifically a drained bulk modulus (Section 2.1.1); here a constant
G	Shear modulus of tissue; constant
k	A loss rate of the free particle or molecule, being a sum of the rates due to capillary loss and to irreversible degradation (which add in the linear approximation). This and rates below are all taken constant
$k_{irr}$	Irreversible endocytosis rate constant of the bound therapeutic particle
$k_{1}$	Linearized binding rate constant of the free particle
$k_{2}$	Linearized unbinding rate constant of the bound particle
*B*	The concentration of binding sites
div, grad	The divergence and gradient operators in vector calculus

**Table 2:** Summary of error measures.

Type of Data	# of Infusions	Bias (%)	L1 Error (%)
MR tracer total volume in MR1-1	5	−22.1	26
MR tracer volume in tumor in MR1-1	5	−2.1	18.3
PET tracer total volume in MR1-1	6	−14	18.96
PET tracer volume in tumor in MR1-1	6	−1.5	17.8
MR tracer total volume in D2C7	23	−9.5	37.6
MR tracer volume in tumor in D2C7	23	3.0	49.6
PET tracer total volume in D2C7	8	−3.8	42.9
PET tracer volume in tumor in D2C7	8	2.8	37

## Abbreviations

ADC (average diffusion coefficient); BBB (blood–brain barrier); CED (convection-enhanced delivery); CSF (cerebrospinal fluid); Da (Daltons); DCE (dynamic contrast-enhanced imaging) (In this work, only MR-DCE (i.e., DCE based on MR imaging) is used, but we omit the “MR” when we refer to it); DTI (diffusion tensor imaging); FA (fractional anisotropy, a measure of directionality in the diffusivity of water); FLAIR (Fluid-attenuated inversion recovery—an MRI sequence used to distinguish edematous tissue from normal fluid-filled regions); Gd or Gad (gadolinium in chelated small molecule form, here as Magnevist™); HSA (human serum albumin); mL (milliliter); mM (millimoles per liter); MRI (magnetic resonance imaging); PET (positron-emission tomography); SPECT (single photon emission computed tomography); VEGF (vascular endothelial growth factor). For nuclear magnetic resonance terms such as T1, T2, TR, and TE, consult any source on magnetic resonance, or the Internet.

## Appendix A. Table of Data for All Infusions

Statistics on all of the infusions that we examined are summarized in Table A1. We give both the distribution volumes and the percentage of coverage of a region, according to the region being the three-dimensional volume of the enhancing region under T1 imaging with Gd contrast administered (endovascularly), the 2-cm margin surrounding this enhancing region, or the total. Every infusion we examined is listed. In the charts given in Section 3.2, only the infusions where the imaging was available for computing both the measured and simulated distributions are shown.

**Table A1:** Error tables: all cases.

CaseID			Distribution Volume (mL)						Coverage (%)				Tumor Volume (mL)	
[n catheters]			Enhancing Tumor		Margin		Total		Enhancing Tumor		Margin		Enhancing	Margin
			*24 h*	*72 h*	*24 h*	*72 h*	*24 h*	*72 h*	*24 h*	*72 h*	*24 h*	*72 h*		
**MR1-1 P07**	Gad	Measured	5.6	6.4	38.2	45.8	48.3	70.4	14.0%	16.0%	22.7%	27.2%	40.0	168.1
[4]		Simulated	6.7	6.7	30.8	37.5	37.7	45.2	16.8%	16.8%	18.3%	22.3%	40.0	168.1
	^124^I	Measured	15.3	21.7	31.3	73.6	50.1	128.6	38.3%	54.3%	18.6%	43.8%	40.0	168.1
		Simulated	22.9	26.4	45.4	70.9	69.5	104.4	57.3%	66.0%	27.0%	42.2%	40.0	168.1
			*24 h*	*54 hr*	*24 h*	*54 hr*	*24 h*	*54 hr*	*24 h*	*54 hr*	*24 h*	*54 hr*		
**MR1-1 P08**	Gad	Measured	4.3	7.0	9.8	16.7	15.4	28.2	7.2%	11.7%	5.3%	9.0%	60.0	186.2
[4]		Simulated	4.6	4.6	11.0	12.0	15.8	17.0	7.7%	7.7%	5.9%	6.4%	60.0	186.2
	^124^I	Measured	29.0	34.5	25.9	64.8	59.5	113.9	48.3%	57.5%	13.9%	34.8%	60.0	186.2
		Simulated	25.7	25.8	26.8	40.2	53.6	69.2	42.8%	43.0%	14.4%	21.6%	60.0	186.2
			*24 h*	*72 h*	*24 h*	*72 h*	*24 h*	*72 h*	*24 h*	*72 h*	*24 h*	*72 h*		
**MR1-1 P09**	Gad	Measured	0.1	0.0	24.2	0.0	29.4	0.0	2.0%	0.0%	45.1%	0.0%	5.0	53.6
[4]		Simulated	0.3	0.3	24.5	25.6	31.8	34.7	6.0%	6.0%	45.7%	47.8%	5.0	53.6
	^124^I	Measured	2.1	2.2	35.2	32.5	47.3	39.4	42.0%	44.0%	65.7%	60.6%	5.0	53.6
		Simulated	1.4	1.4	28.3	30.3	37.8	42.9	28.0%	28.0%	52.8%	56.5%	5.0	53.6
			*24 h*	*72 h*	*24 h*	*72 h*	*24 h*	*72 h*	*24 h*	*72 h*	*24 h*	*72 h*		
**D2C7 P1001**	Gad	Measured	1.0		0.6		1.9	0.0	3.3%	0.0%	0.6%	0.0%	30.1	95.0
[2]		Simulated	4.2	4.2	1.7	1.7	5.9	5.9	14.0%	14.0%	1.8%	1.8%	30.1	95.0
	^124^I	Simulated	20.4	26.2	12.9	31.1	33.3	57.3	67.8%	87.0%	13.6%	32.7%	30.1	95.0
			*24 h*	*72 h*	*24 h*	*72 h*	*24 h*	*72 h*	*24 h*	*72 h*	*24 h*	*72 h*		
**D2C7 P1002**	Gad	Measured	1.5	2.0	5.3	4.0	6.8	6.0	15.0%	20.0%	6.2%	4.7%	10.0	86.0
[1]		Simulated	2.9	2.9	4.2	4.2	7.1	7.1	29.0%	29.0%	4.9%	4.9%	10.0	86.0
	^124^I	Measured	4.7	3.3	18.1	25.8	22.8	29.1	47.0%	33.0%	21.0%	30.0%	10.0	86.0
		Simulated	5.5	7.5	11.2	19.6	16.7	27.1	55.0%	75.0%	13.0%	22.8%	10.0	86.0
			*24 h*	*72 h*	*24 h*	*72 h*	*24 h*	*72 h*	*24 h*	*72 h*	*24 h*	*72 h*		
**D2C7 P1004**	Gad	Measured	0.8	1.5	0.1	0.1	0.8	1.6	9.9%	19.0%	0.1%	0.1%	7.9	81.0
[1]		Simulated	1.2	1.2	0.2	0.2	1.4	1.4	15.2%	15.2%	0.2%	0.2%	7.9	81.0
	^124^I	Measured	4.6	7.7	1.2	11.3	5.8	20.8	58.2%	97.0%	1.5%	14.0%	7.9	81.0
		Simulated	7.4	7.4	11.3	21.9	20.0	32.0	93.0%	94.1%	14.0%	27.0%	7.9	81.0
			*24 h*	*72 h*	*24 h*	*72 h*	*24 h*	*72 h*	*24 h*	*72 h*	*24 h*	*72 h*		
**D2C7 P1005**	Gad	Measured	0.7	0.1	1.5	0.2	2.6	0.4	10.0%	1.5%	1.6%	0.2%	6.6	96.0
[1]		Simulated	0.6	0.6	0.5	0.5	1.1	1.1	9.1%	9.1%	0.5%	0.5%	6.6	96.0
	^124^I	Measured	4.9	5.0	9.1	14.4	14.0	19.4	74.2%	75.8%	9.5%	15.0%	6.6	96.0
		Simulated	2.4	2.9	3.9	6.1	6.3	9.0	36.4%	43.9%	4.1%	6.4%	6.6	96.0
			*24 h*	*72 h*	*24 h*	*72 h*	*24 h*	*72 h*	*24 h*	*72 h*	*24 h*	*72 h*		
**D2C7 P1006**	Gad	Measured	0.6	1.2	0.2	0.4	0.8	1.6	7.2%	14.5%	0.2%	0.4%	8.3	94.3
[1]		Simulated	0.5	0.5	0.1	0.1	0.6	0.6	5.4%	5.4%	0.1%	0.1%	8.3	94.3
	^124^I	Measured	4.8	4.2	3.8	4.0	9.2	8.9	57.8%	50.6%	4.0%	4.2%	8.3	94.3
		Simulated	3.3	3.9	2.3	3.8	5.6	7.7	39.8%	47.0%	2.4%	4.0%	8.3	94.3
			*24 h*	*72 h*	*24 h*	*72 h*	*24 h*	*72 h*	*24 h*	*72 h*	*24 h*	*72 h*		
**D2C7 P1007**	Gad	Measured	1.0	0.2	2.9	1.8	3.9	2.0	26.3%	5.3%	4.9%	3.1%	3.8	59.0
[1]		Simulated	0.7	0.7	2.0	2.0	2.7	2.7	18.4%	18.4%	3.4%	3.4%	3.8	59.0
	^124^I	Simulated	2.4	3.2	7.7	10.6	10.1	13.8	63.2%	84.2%	13.1%	18.0%	3.8	59.0
			*24 h*	*72 h*	*24 h*	*72 h*	*24 h*	*72 h*	*24 h*	*72 h*	*24 h*	*72 h*		
**D2C7 P1008**	Gad	Measured	1.6	1.5	0.8	1.2	2.4	2.7	26.7%	25.0%	1.6%	2.4%	6.0	50.0
[1]														
			*24 h*	*72 h*	*24 h*	*72 h*	*24 h*	*72 h*	*24 h*	*72 h*	*24 h*	*72 h*		
**D2C7 P1025**	Gad	Measured		0.10		1.44	0.00	1.90	0.0%	7.2%	0.0%	3.0%	1.4	47.4
[1]		Simulated	0.16	0.16	2.36	2.37	2.52	2.53	11.6%	11.6%	5.0%	5.0%	1.4	47.4
	^124^I	Simulated	0.97	0.98	5.77	6.79	6.74	7.82	70.3%	71.0%	12.2%	14.3%	1.4	47.4
			*24 h*	*72 h*	*24 h*	*72 h*	*24 h*	*72 h*	*24 h*	*72 h*	*24 h*	*72 h*		
**D2C7 P1026**	Gad	Measured	0.08	0.11	1.13	2.84	1.21	2.95	1.0%	1.3%	1.9%	4.8%	8.2	59.5
[1]		Simulated	0.45	0.45	0.80	0.84	1.32	1.36	5.5%	5.5%	1.3%	1.4%	8.2	59.5
	^124^I	Simulated	6.66	6.80	9.48	11.14	17.06	18.86	80.9%	82.6%	15.9%	18.7%	8.2	59.5
			*24 h*	*72 h*	*24 h*	*72 h*	*24 h*	*72 h*	*24 h*	*72 h*	*24 h*	*72 h*		
**D2C7 P1031**	Gad	Measured	0.45	0.22	0.05	0.36	0.80	0.70	22.2%	10.8%	0.1%	0.7%	2.0	50.2
[1]		Simulated	0.50	0.50	0.40	0.40	1.24	1.24	24.6%	24.6%	0.8%	0.8%	2.0	50.2
	^124^I	Simulated	1.96	1.97	10.44	14.08	12.82	16.52	96.6%	97.0%	20.8%	28.0%	2.0	50.2
			*24 h*	*72 h*	*24 h*	*72 h*	*24 h*	*72 h*	*24 h*	*72 h*	*24 h*	*72 h*		
**D2C7 P1032**	Gad	Measured	0.64	1.08	0.55	1.03	1.25	2.11	25.3%	42.7%	1.3%	2.3%	2.5	43.9
[1]		Simulated	1.45	1.45	1.92	1.96	3.50	3.50	57.3%	57.3%	4.4%	4.5%	2.5	43.9
	^124^I	Simulated	2.53	2.53	10.10	15.05	12.63	17.58	100.0%	100.0%	23.0%	34.3%	2.5	43.9
			*24 h*	*72 h*	*24 h*	*72 h*	*24 h*	*72 h*	*24 h*	*72 h*	*24 h*	*72 h*		
**D2C7 P1034**	Gad	Measured	0.99	1.75	0.19	0.95	1.96	2.90	4.2%	7.4%	0.2%	0.9%	23.7	102.0
[1]		Simulated	1.48	1.48	0.00	0.00	1.86	1.86	6.2%	6.2%	0.0%	0.0%	23.7	102.0
	^124^I	Simulated	15.73	22.94	2.06	9.54	18.79	33.71	66.4%	96.8%	2.0%	9.4%	23.7	102.0
			*24 h*	*72 h*	*24 h*	*72 h*	*24 h*	*72 h*	*24 h*	*72 h*	*24 h*	*72 h*		
**D2C7 P1037**	Gad	Measured	1.90	1.73	0.61	0.44	2.65	2.26	34.5%	31.5%	0.9%	0.6%	5.5	70.2
[1]		Simulated	1.53	1.53	0.27	0.27	1.80	1.80	27.8%	27.8%	0.4%	0.4%	5.5	70.2
	^124^I	Simulated	3.46	4.16	2.88	5.18	6.41	9.39	62.9%	75.6%	4.1%	7.4%	5.5	70.2
			*24 h*	*72 h*	*24 h*	*72 h*	*24 h*	*72 h*	*24 h*	*72 h*	*24 h*	*72 h*		
**D2C7 P1041**	Gad	Measured	1.61	4.78	3.02	9.52	4.74	15.41	6.2%	18.4%	1.6%	5.0%	26.0	191.0
[2]		Simulated	1.46	1.46	3.21	3.49	5.44	5.71	5.6%	5.6%	1.7%	1.8%	26.0	191.0
	^124^I	Simulated	15.76	20.89	36.04	76.69	54.50	102.65	60.6%	80.3%	18.9%	40.2%	26.0	191.0

## Appendix B. Potential Applications of Models of Flow and Transport

We list a number of applications which we may envisage for the methods described in this paper, allowing, in some cases, extensions that we have not yet developed.

**Table A2:** Potential uses of modeling.

Model/Simulate/Predict:	Application:
Resting hydraulic conductivity of tissue	Plan catheter placement surgeries
Extracellular volume increase under forced infusion	Plan safe convection-enhanced delivery
Convective transport of macromolecules	Direct drug delivery planning
Convective transport of viral delivery agents	Viral vector delivery planning
Intra-cerebral pressure distributions, normal brain	Baseline patient-specific atlas
Endogenous bulk flow pathways in normal brain	Predict deposition routes for plaque etc.
Intra-cerebral pressure distributions, edematous brain	Plan chemotherapies, direct drug delivery
Endogenous bulk flow pathways in edematous brain	Pathways map for cell and other transport
Intra-cerebral pressure across trauma and other brain insults	Treat and stage brain insults and injuries
Convective (passive) transport of cells	Likely sites of glioblastoma recurrence
Distance interactions and signals for cell migration	Planning stem cell delivery and migration
Glioblastoma cells: proliferation, bulk flow, diffusion	Stage/treat disease, plan radiotherapy, etc.
Backflow from cylindrical catheters	Catheter design and placement
Backflow from novel catheters (stepped, spherical, *etc.)*	Other device design, e.g., balloons in tissue
Tissue mechanics and response to pressure	Plan neurosurgery allowing brain shift, etc.
Tissue response to mechanics and ultrasound	Enhance penetration of CSF–brain barriers
Streaming under ultrasound	Enhance drug delivery
Tissue mechanics with cell growth	Cancer cell proliferation;
Small and macro molecules across the vascular barrier	Plan dosage for CNS applications
Transport + metabolism + kinetics using receptor densities	Plan dosage for drugs with toxicity
